# Using Hypothesis-Led Machine Learning and Hierarchical Cluster Analysis to Identify Disease Pathways Prior to Dementia: Longitudinal Cohort Study

**DOI:** 10.2196/41858

**Published:** 2023-07-26

**Authors:** Shih-Tsung Huang, Fei-Yuan Hsiao, Tsung-Hsien Tsai, Pei-Jung Chen, Li-Ning Peng, Liang-Kung Chen

**Affiliations:** 1 Department of Pharmacy National Yang Ming Chiao Tung University Taipei Taiwan; 2 Center for Healthy Longevity and Aging Sciences National Yang Ming Chiao Tung University Taipei Taiwan; 3 Graduate Institute of Clinical Pharmacy, College of Medicine National Taiwan University Taipei Taiwan; 4 School of Pharmacy College of Medicine National Taiwan University Taipei Taiwan; 5 Department of Pharmacy National Taiwan University Hospital Taipei Taiwan; 6 Advanced Tech Business Unit Acer New Taipei City Taiwan; 7 Center for Geriatrics and Gerontology Taipei Veterans General Hospital Taipei Taiwan; 8 Taipei Municipal Gan-Dau Hospital (Managed by Taipei Veterans General Hospital) Taipei Taiwan

**Keywords:** dementia, machine learning, cluster analysis, disease, condition, symptoms, data, data set, cardiovascular, neuropsychiatric, infection, mobility, mental conditions, development

## Abstract

**Background:**

Dementia development is a complex process in which the occurrence and sequential relationships of different diseases or conditions may construct specific patterns leading to incident dementia.

**Objective:**

This study aimed to identify patterns of disease or symptom clusters and their sequences prior to incident dementia using a novel approach incorporating machine learning methods.

**Methods:**

Using Taiwan’s National Health Insurance Research Database, data from 15,700 older people with dementia and 15,700 nondementia controls matched on age, sex, and index year (n=10,466, 67% for the training data set and n=5234, 33% for the testing data set) were retrieved for analysis. Using machine learning methods to capture specific hierarchical disease triplet clusters prior to dementia, we designed a study algorithm with four steps: (1) data preprocessing, (2) disease or symptom pathway selection, (3) model construction and optimization, and (4) data visualization.

**Results:**

Among 15,700 identified older people with dementia, 10,466 and 5234 subjects were randomly assigned to the training and testing data sets, and 6215 hierarchical disease triplet clusters with positive correlations with dementia onset were identified. We subsequently generated 19,438 features to construct prediction models, and the model with the best performance was support vector machine (SVM) with the by-group LASSO (least absolute shrinkage and selection operator) regression method (total corresponding features=2513; accuracy=0.615; sensitivity=0.607; specificity=0.622; positive predictive value=0.612; negative predictive value=0.619; area under the curve=0.639). In total, this study captured 49 hierarchical disease triplet clusters related to dementia development, and the most characteristic patterns leading to incident dementia started with cardiovascular conditions (mainly hypertension), cerebrovascular disease, mobility disorders, or infections, followed by neuropsychiatric conditions.

**Conclusions:**

Dementia development in the real world is an intricate process involving various diseases or conditions, their co-occurrence, and sequential relationships. Using a machine learning approach, we identified 49 hierarchical disease triplet clusters with leading roles (cardio- or cerebrovascular disease) and supporting roles (mental conditions, locomotion difficulties, infections, and nonspecific neurological conditions) in dementia development. Further studies using data from other countries are needed to validate the prediction algorithms for dementia development, allowing the development of comprehensive strategies to prevent or care for dementia in the real world.

## Introduction

The 2015 World Health Organization (WHO) estimate indicated that the population of people with dementia, currently at 47 million, will triple by 2050 due to global population aging [1]. Asia, as the most populated continent with a rapid population aging speed [2], is expected to experience exponential growth in the population that contains people with dementia. Due to the lack of a cure for dementia, current public health and clinical efforts are mainly focused on early detection, timely diagnosis, and prevention of dementia. Several well-designed clinical trials have validated the potential to prevent age-related cognitive decline among community-dwelling older adults through multidomain intervention programs [3,4]. Hence, early identification of older people at risk for dementia has gained extensive attention in the effort to prevent or delay the onset of dementia.

Dementia, a progressive neurodegenerative disorder, is closely linked with several risk factors [5], including those with modifiable properties [6]. Various modifiable risk factors have been identified as contributing to dementia development, such as cardiometabolic risk, physical activity [7,8], depressive symptoms [9], use of psychotropic drugs [10,11], medications with anticholinergic burden [12,13], and *Helicobacter pylori* infection [14]. Previous studies have constructed comprehensive prediction models that cover these risk factors and multiple dimensions related to dementia development, particularly imaging data, to provide guidance for prevention strategies [15,16]. However, most of these prediction models are more suitable for longitudinal research settings than for clinical practice, and the evidence supporting the effects of intervening on the risk factors in the prediction model is limited [17,18].

In addition, while these factors can be treated separately, they may be interconnected. For instance, the increased use of psychotropic drugs before dementia may be a result of prodromal behavioral and psychiatric symptoms rather than medication effects [19]. Thus, it is essential to recognize the potential “cluster and sequelae” pattern of these risk factors before diagnosing dementia instead of solely focusing on individual risk factors. Moreover, most prediction models for dementia rely heavily on features extracted from imaging data. While models incorporating imaging data achieve higher accuracy, clinical patients do not routinely undergo brain imaging tests, especially when there are no symptoms of suspected dementia [20,21]. Therefore, while these models provide good accuracy at the research level, they have limited value in the early detection of patients at high risk of developing dementia in clinical settings.

To overcome the limitations of this literature, our hypothesis posits that the occurrence of specific disease patterns or clusters of symptoms, as well as their sequential development preceding the onset of dementia, greatly elevates the risk of developing this condition. Notably, medical records and claims data readily provide information on these symptoms and diseases, which could potentially facilitate clinical applications for preventing dementia in the real world. Nonetheless, identifying disease pathways prior to dementia diagnosis through conventional epidemiological methods and statistical models poses a complex clinical challenge. Hence, machine learning has emerged as a valuable tool for detecting disease pathways. By offering objective classification criteria, machine learning models enhance the reliability and validity of assessments. Recent advances in machine learning techniques show promise in identifying disease clusters and analyzing longitudinal data. For instance, previous studies have used unsupervised learning algorithms, like hierarchical clustering, to classify patients based on gene expression data and recognize disease subtypes [22,23]. Furthermore, supervised learning algorithms, such as LASSO (least absolute shrinkage and selection operator)regression, support vector machines (SVM), and random forests, have been used to predict disease progression and pinpoint potential biomarkers [24-27].

Therefore, this study aimed to use nationwide claims data from Taiwan’s National Health Insurance to identify patterns of disease or symptom clusters and their sequences prior to incident dementia using a novel approach incorporating machine learning methods to identify at-risk patterns of disease or symptom clusters and their sequences for preventive intervention activities.

## Methods

### Study Design and Participants

This is a retrospective cohort study using data from Taiwan's National Health Insurance Research Database (NHIRD). The details of the NHIRD have been published in previous studies [28]. The NHIRD is a nationwide database composed of outpatient and inpatient claims covering more than 99% of Taiwan's inhabitants (approximately 24 million in 2019). The Data Science Center of the Ministry of Health and Welfare, Taiwan, regularly performed data quality checks and related maintenance. We used a subset of the NHIRD, which contains claims data for 1 million randomly selected beneficiaries from the Registry of Beneficiaries of the NHIRD in 2005. We identified patients aged 55 years and older who were first diagnosed with dementia (defined as receiving a diagnosis of International Classification of Disease, 9th Edition codes 290.xx, 294.xx, or 331.xx) from January 1, 2003, to December 31, 2013. The dementia diagnosis was considered valid if it was documented at least 3 times in outpatient visits within 1 year or at any admission. The index date was defined as the date of first diagnosis of dementia. To examine the specific patterns of disease or symptom clusters and sequences prior to the diagnosis of dementia, the data retrieval period for each study subject started on the index date and was traced back to January 1, 2003.

### Ethics Approval

The study protocol was approved by the Research Ethics Committee of the National Taiwan University Hospital (NTUH-REC-201803134RINC).

### Study Design

Data from people with dementia were randomized into the training data set (two-thirds of all cases) and the testing data set (the remaining one-third). Furthermore, 1 matched control of a person without dementia was randomly assigned to each person with dementia in both the training and testing data sets according to age, sex, and index year. To examine the potential disease or symptom patterns associated with the onset of dementia using machine learning methods, we designed a study algorithm with four steps: (1) data preprocessing, (2) disease or symptom pattern selection, (3) model construction and optimization, and (4) data visualization.

### Model Construction

**Figure 1:**
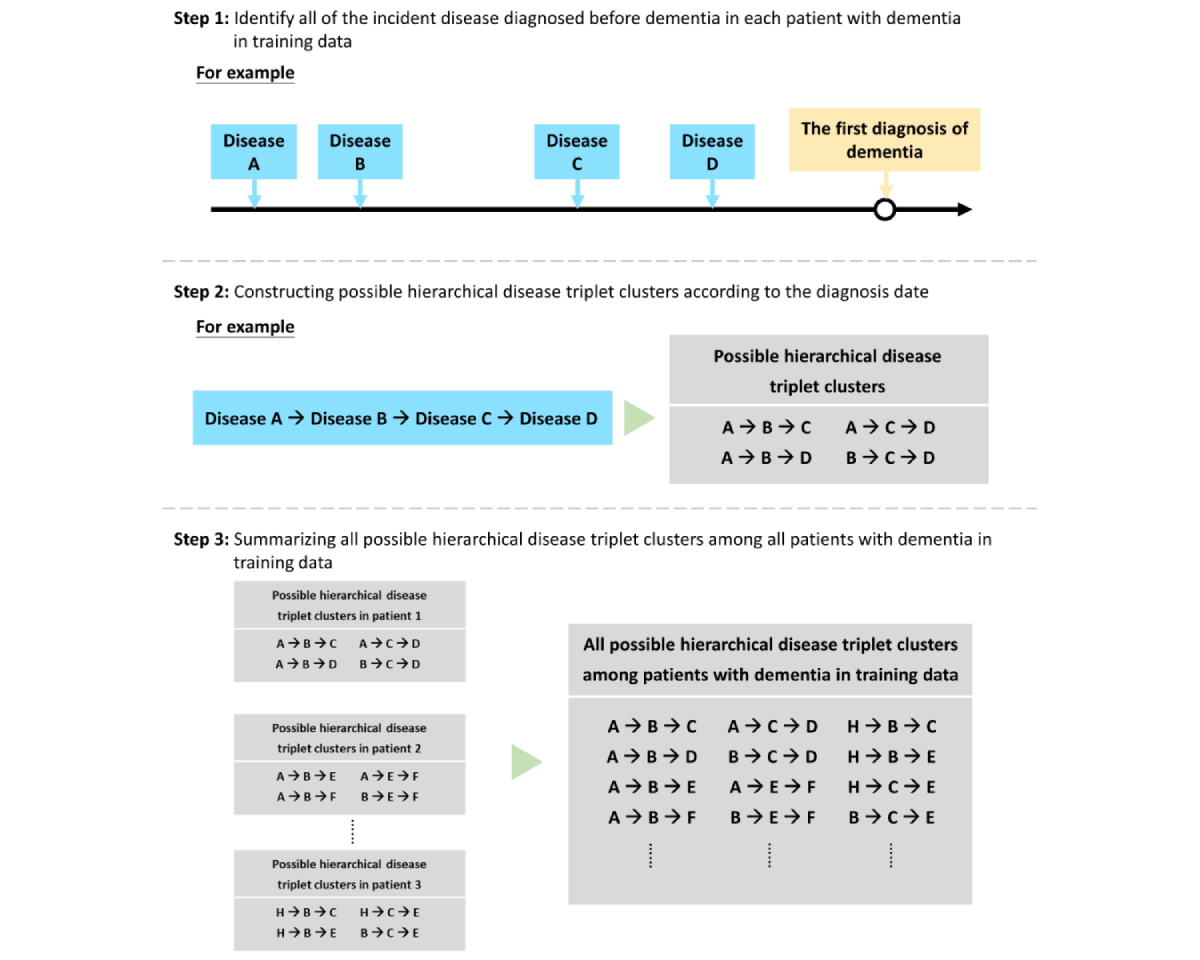
Data preprocessing.

First, we identified all incident disease diagnoses before the index date for each person with dementia in the training data set using Clinical Classification Software codes [29], a diagnosis coding system collapsing over 14,000 International Classification of Disease, 9th Edition codes into 253 categories that are more applicable to health data analysis. The disease diagnosis was considered valid if it was documented at least 3 times in outpatient visits within 1 year or 1 admission record. The diagnosis date was defined as the date of first diagnosis of each disease. For data preprocessing, we sorted the diseases in sequences according to the diagnosis date and constructed all possible hierarchical disease triplet clusters (Figure 1). Older adults in Taiwan made approximately 30 outpatient visits a year and had a significant number of diagnoses documented in the NHIRD [30], which provide abundant records of disease diagnoses. In this study, we focused on hierarchical disease triplet clusters to reduce data complexity.

**Figure 2:**
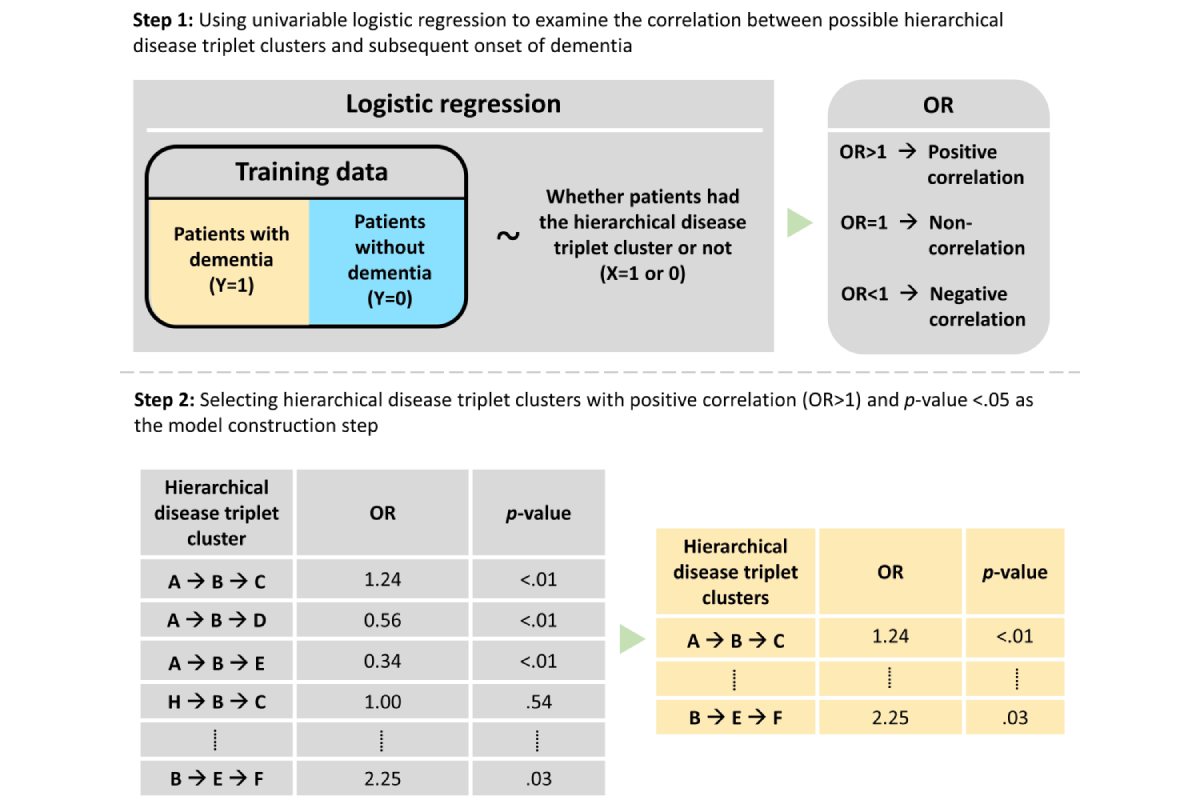
Selecting hierarchical disease triplet clusters with positive associations. OR: odds ratio.

After identifying all possible hierarchical disease triplet clusters in people with dementia, we used univariable logistic regression to examine the correlations between every potential hierarchical disease triplet cluster and the subsequent onset of dementia. Only the hierarchical disease triplet clusters with a positive correlation (odds ratio >1) and *P* values <.05 were selected for the logistic regression model at the model construction step (Figure 2).

**Figure 3:**
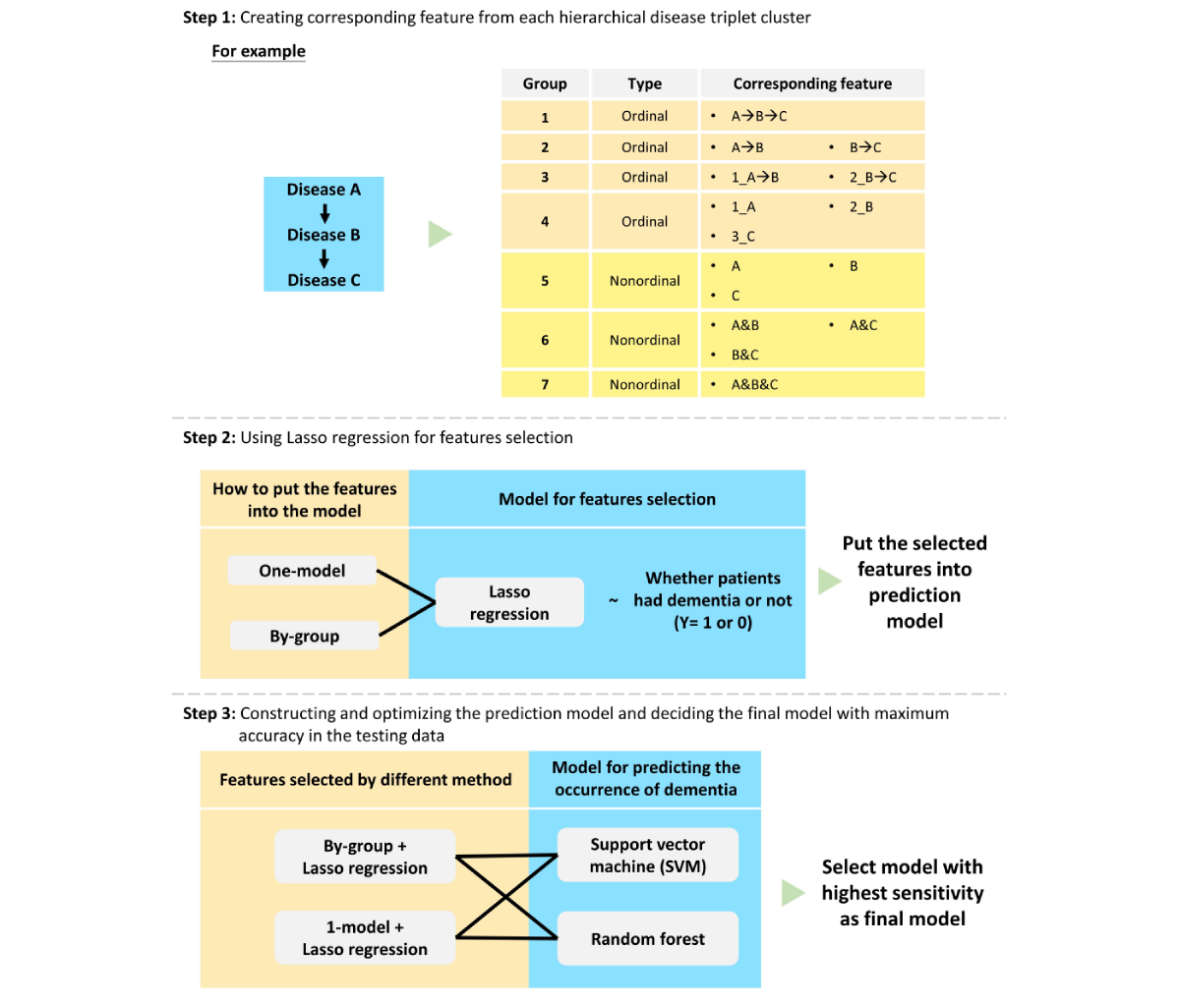
Model construction and optimization.

Before model construction, we created corresponding features from each hierarchical disease triplet cluster to extract the information contained in the clusters. In our study, we generated 7 different corresponding features from the original hierarchical disease triplet clusters, including 4 ordinal and 3 nonordinal corresponding features. Table 1 summarizes the details regarding the definition and explanation of the corresponding features. Then, we used multivariable LASSO regression collocated with a 1-model (put all the corresponding features into the model) or by-group method (put corresponding features into the model separately according to the categories of corresponding features and then identify the union of selected features between each model). In this study, we constructed 2 methods for feature selection, a process that selected and filtered significant variables used in the prediction model. Last, using the corresponding features selected by the 2 abovementioned methods, we constructed SVM or random forest classifiers with 3-fold cross-validation for each method and yielded 4 machine learning models to predict subsequent dementia. We chose the model with the best sensitivity in the testing data set as our final model (Figure 3).

**Table 1:** Definitions of the corresponding features generated in this study.

Group	Type	Example of the corresponding feature	Explanation
1	Ordinal	A→B→C	The feature of the original hierarchical disease triplet clusters Example: A→B→C means that disease A, disease B, and disease C occurred sequentially in the pathway
2	Ordinal	A→B B→C	The feature of the ordered disease doublets in the hierarchical disease triplet clusters Example: A→B means that disease A and disease B occurred sequentially in the hierarchical disease triplet clusters (disease A can be the first or second disease in the cluster)
3	Ordinal	1_A→B 2_B→C	The feature of ordered disease doublets with a specific position in the hierarchical disease triplet clusters Example: 1_A→B means that disease A and disease B occurred sequentially, and disease A is the first disease in the hierarchical disease triplet clusters
4	Ordinal	1_A 2_B 3_C	The feature of a single disease with a specific position in the hierarchical disease triplet clusters Example: 1_A means that disease A is the first disease in the hierarchical disease triplet clusters
5	Nonordinal	A B C	The feature of a single disease in the hierarchical disease triplet clusters Example: A means that disease A is in the hierarchical disease triplet cluster, regardless of the order
6	Nonordinal	A&B A&C B&C	The feature of disease doublets in the hierarchical disease triplet clusters Example: A&B means that disease A and disease B are in the hierarchical disease triplet clusters, regardless of the order
7	Nonordinal	A&B&C	The feature of disease triplets in the hierarchical disease triplet clusters Example: A&B&C means that disease A, disease B, and disease C are in the hierarchical disease triplet clusters, regardless of the order

### Disease Tree and Temporal Disease Network

As a result of the multitude of disease pathways identified in the final model, presenting the results became a challenging task. To overcome this issue, we used a disease tree method that involved consolidating common ordinal corresponding features within each pathway into nodes. The parent node represented the highest level of the pathway, while intermediate levels were referred to as children nodes. However, given the vast number of parent nodes, identifying clinically meaningful pathways proved to be difficult. Thus, we used the cumulative covered dementia population fraction approach to determine the significance of parent nodes and their hierarchical disease triplet clusters. This approach gauged how many parent nodes were included in the final model to provide the marginally highest model accuracy. Our findings indicated that 49 parent nodes were the optimal number when the covering accuracy reached its highest level. To present the results succinctly, we selected the top 5 children nodes with the highest dementia probabilities for each parent node, indicating a higher proportion of individuals within these pathways are likely to develop dementia. This approach enabled us to focus on the most significant and relevant pathways associated with the risk of dementia.

**Figure 4:**
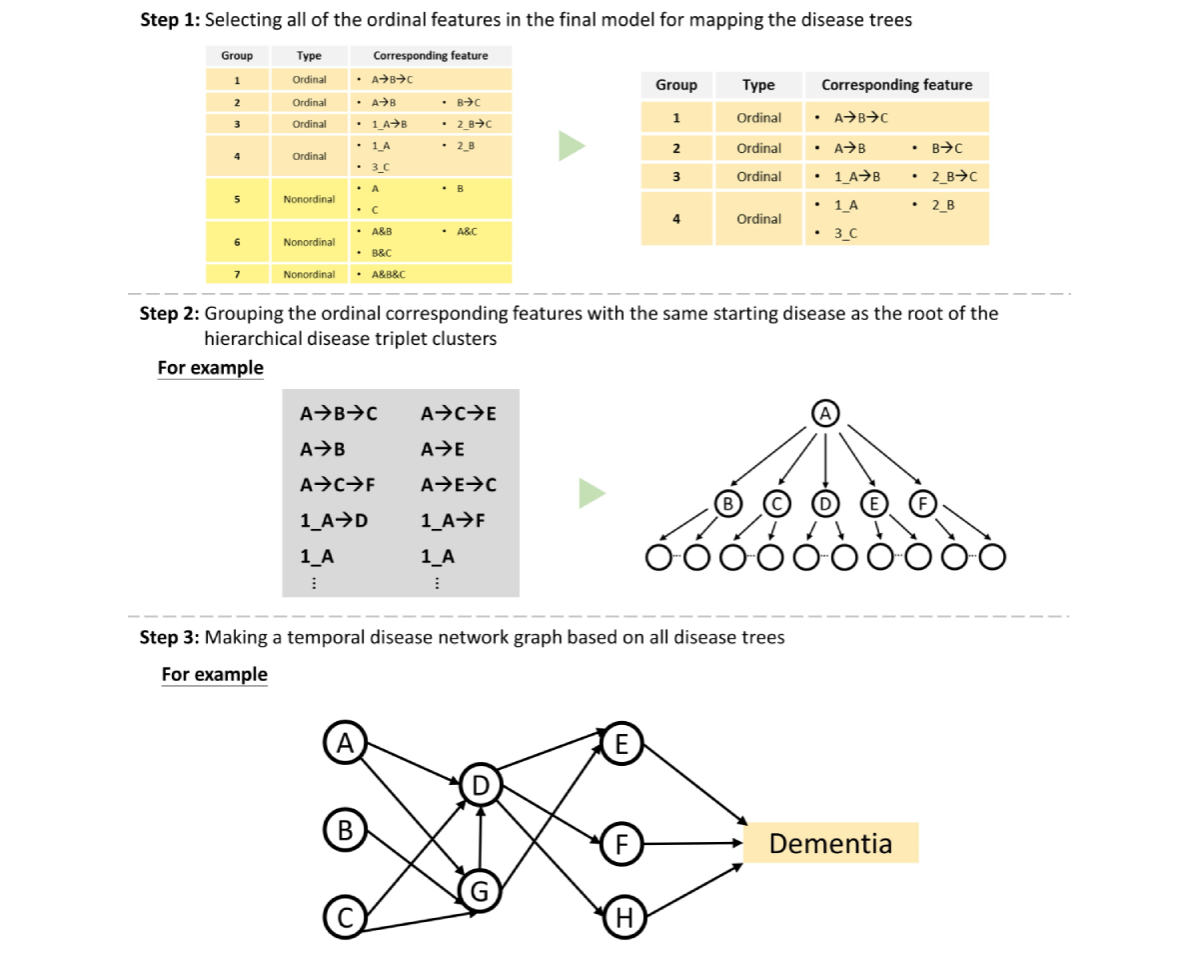
Data visualization.

Moreover, to provide an overall picture of the parent nodes and the relevant disease pathway, we subsequently made a temporal disease network graph based on all disease trees, whereby edges linking directed disease pairs show how frequently alternative disease paths are followed over time. To further simplify the temporal disease network graph, we showed only pathways with at least 50 patients with dementia (Figure 4).

### Statistical Analysis

All analyses were performed using R (version 3.6.2; R Foundation for Statistical Computing) with a publicly available package. We used the *glmnet_2.0-16*, *e1071_1.7-9*, *randomForest_4.6-14*, and *stats_3.6.2* packages for model construction. A 2-sided *P* value of <.05 was considered statistically significant.

## Results

### Model Construction

In this study, we identified 15,700 people with dementia, and 10,466 of them were randomly assigned into the training data set with the remaining 5234 people with dementia in the testing data set. Among the people with dementia in the training data set, 618,249 potential hierarchical disease triplet clusters (pathways) containing at least 10 people were identified, and 6215 hierarchical disease triplet clusters with a positive correlation with dementia onset were selected by logistic regression. We further generated 19,438 corresponding features for model construction. Table 2 summarizes the results of the model selection. The final model with the highest model sensitivity was the SVM model with the by-group LASSO regression method (total corresponding features=2513; accuracy=0.615; sensitivity=0.607; specificity=0.622; positive predictive value=0.612; negative predictive value=0.619; area under the curve=0.639).

**Table 2:** Results of the model selection.

Method of feature selection (number of features)	Model	Accuracy, %	Sensitivity, %	Specificity, %	PPV^a^, %	NPV^b^, %	AUC^c^, %
By-group LASSO (n=2513)	SVM^d^	61.5	60.7	62.2	61.2	61.9	63.9
1-model LASSO (n=181)	SVM	61.9	57.9	65.9	62.4	61.6	62.6
By-group LASSO (n=2513)	Random forest	61.3	50.4	72.2	63.9	59.9	64.0
1-Model LASSO (n=181)	Random forest	61.8	48.3	75.5	65.6	59.8	64.7

^a^PPV: positive predictive value.

^b^NPV: negative predictive value.

^c^AUC: area under the curve.

^d^SVM: support vector machine.

### Disease Tree and Temporal Disease Network

**Figure 5:**
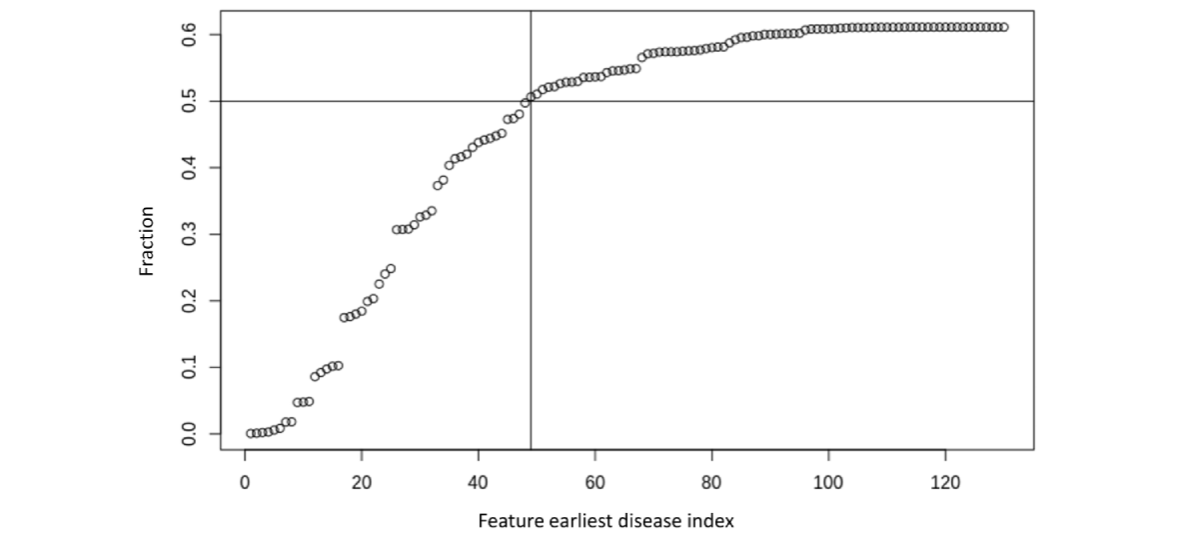
Cumulative covered dementia population fraction in the final model.

We mapped 49 hierarchical disease triplet clusters with distinct roots according to the cumulative covered dementia population fraction (Figure 5). These hierarchical disease triplet clusters covered approximately 50% of all people with dementia in the testing data set. To better visualize disease trees, we showed only the top 5 subtrees and leaves, which covered the highest number of people with dementia.

**Figure 6:**
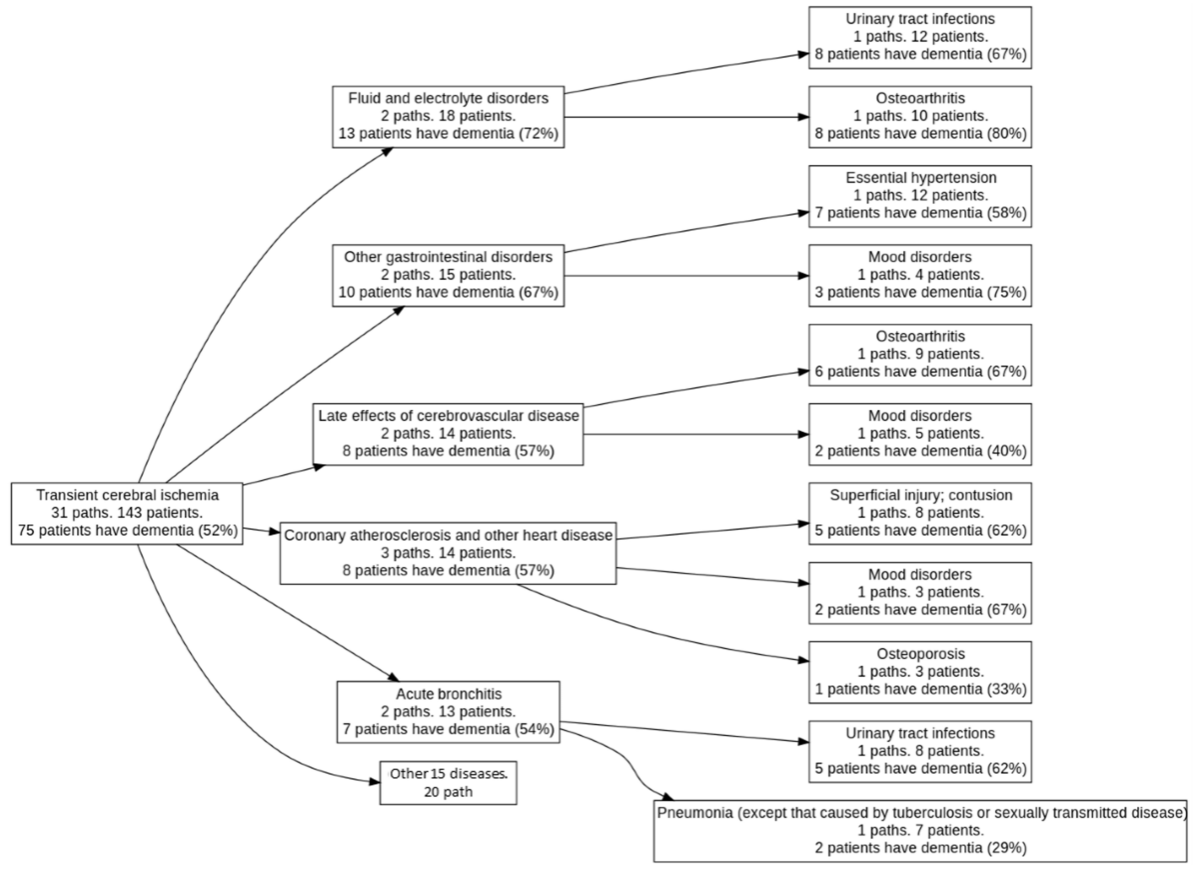
Hierarchical disease triplet clusters starting from transient cerebral ischemia.

**Figure 7:**
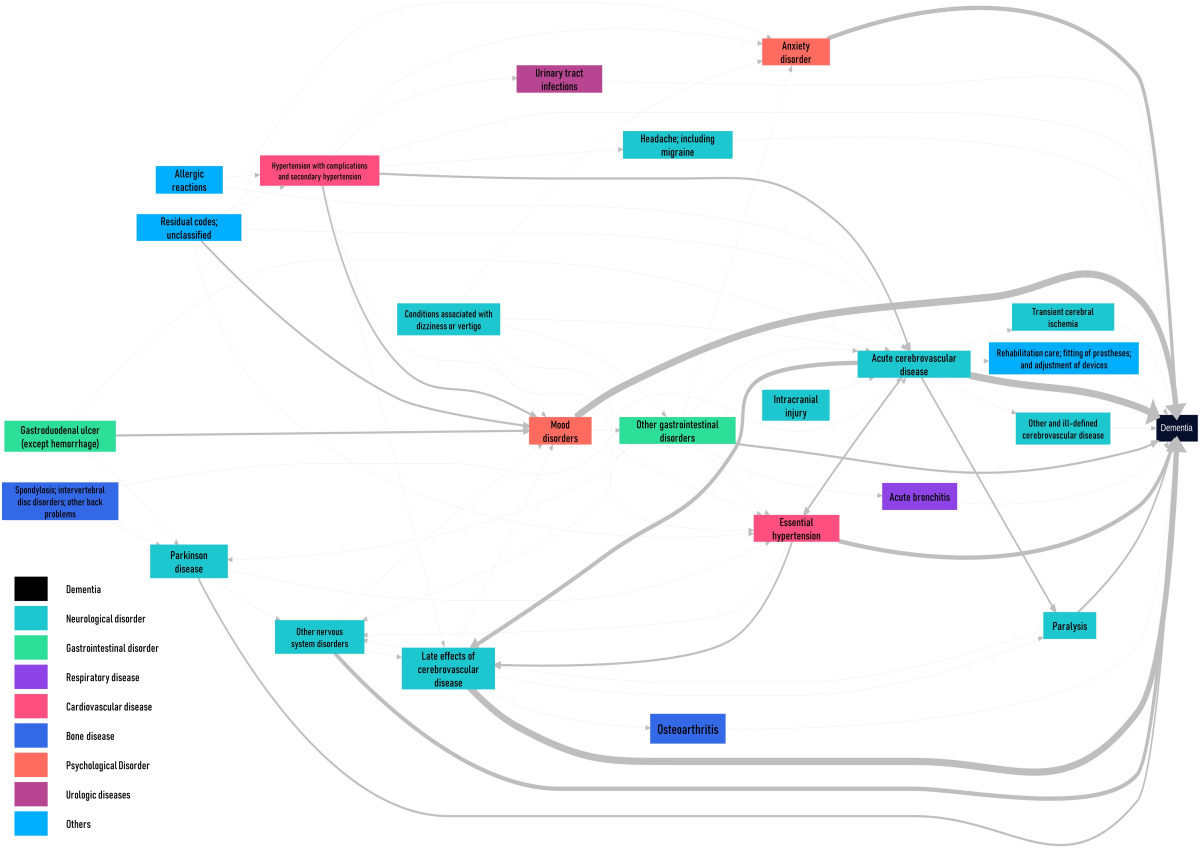
Temporal disease network graph of the diseases before dementia. The thickness of the edges linking diseases indicates how frequently a particular disease path is followed over time. Only pathways containing more than 50 patients with dementia are shown in the network. Diseases are colored according to the disease category.

Figure 6 shows an example of the hierarchical disease triplet clusters of transient cerebral ischemia, followed by coronary atherosclerosis or other heart diseases and then mood disorders to dementia. The other 48 hierarchical disease triplet clusters are presented in Figure S1 in Multimedia Appendix 1. All hierarchical disease triplet clusters can be further aggregated into a disease progression network that shows the overall picture of possible pathways related to dementia development. In this study, many hierarchical disease triplet clusters included acute cerebrovascular disease, mood disorders, late effect of cerebrovascular disease, other nervous system disorders, anxiety disorders, essential hypertension, Parkinson disease, and other gastrointestinal disorders (Figure 7).

## Discussion

### Principal Findings

To the best of our knowledge, this is the first study incorporating machine learning methods with an established hypothesis and using national health insurance claims data to generate potential hierarchical disease triplet clusters (disease trees) leading to incident dementia, which is beneficial in health care systems to identify people at risk for dementia via their disease or symptom patterns.

### Comparison to Prior Work

Reinke et al [25] used machine learning techniques, including logistic regression, gradient boosting, and random forests, to construct a prediction model for dementia. Their findings were consistent with our study results, indicating cerebrovascular disease and cardiovascular diseases as highly significant features in the model. However, Nori et al [24], who used the LASSO algorithm to establish a prediction model of dementia, suggested that memory loss, agitation, mild cognitive decline, and bipolar disorder played the most crucial role in the model. In contrast, cerebrovascular disease and cardiovascular diseases only had mild importance in the model. The differences between the 2 studies could be attributed to the varying ways in which disease was defined over time. Reinke et al [25] recorded disease status as a time-varying variable with quarterly measurement, giving more weight to diseases that occurred earlier and persisted. On the other hand, Nori et al [24] defined disease occurrence within 1 to 730 days prior to dementia diagnosis, indicating that most of the features of the disease with high importance in the model may be related to prodromal symptoms of dementia, such as cognitive decline and agitation, while other chronic disease features may have a lower contribution in the model. These discrepancies underscore the importance of incorporating epidemiological concepts into machine learning study design to achieve more causally relevant findings.

In our study, we used several epidemiological concepts to bolster the causal inference of the disease pathway identified in the final model. First, we ensured that all diseases analyzed occurred prior to the dementia diagnosis. This criterion was crucial in establishing temporal precedence, a critical prerequisite for establishing a causal relationship. Second, we used the incidence new user design, which is widely regarded as the gold standard in epidemiological studies for establishing causal relationships. By restricting our analysis to incident cases of dementia instead of prevalent dementia cases, we mitigated the bias risk associated with prevalent cases. Finally, we advanced beyond merely clustering triple diseases and instead sequenced the diseases based on their diagnosis date. This enabled us to incorporate the concept of time series into our model and achieve results with improved causal inference. Collectively, these measures bolstered the causal inference of our model, providing a robust foundation for subsequent machine learning approaches in identifying disease pathways prior to dementia.

Among the identified hierarchical disease triplet clusters, the most common leading diagnoses were cerebrovascular disease (acute or late effect) and hypertension (essential or with complications), which are compatible with the current understanding that cardiometabolic risk appears early in the preclinical stage of dementia. Other characteristic diseases in the leaves included other cardiometabolic conditions, infections (acute bronchitis and urinary tract infection), musculoskeletal conditions (osteoarthritis and spondylosis), gastrointestinal conditions, and neuropsychiatric conditions (mood and anxiety disorders). Overall, the results of this study suggested a characteristic pathway from cardiometabolic diseases (mainly hypertension), cerebrovascular disease, mobility disorders, infections, or neuropsychiatric conditions to incident dementia. Currently, no evidence supports mass neuropsychological screening for dementia, but timely diagnosis for dementia has been prioritized to initiate care planning and interventions for at-risk populations. Previous epidemiological studies have clearly shown associations between cardiometabolic risk and incident dementia, so appropriate cardiometabolic risk management with a life-course approach has become the fundamental element of dementia prevention. Recent randomized controlled trials have confirmed the clinical benefits of multidomain interventions in reducing the risk of cognitive decline [4,31,32]. Through integrated programs incorporating exercise, cognitive training, dietary counseling, and chronic condition management, cardiometabolic risk, locomotion, depressive symptoms, and nutrition were improved, which may attenuate the dementia risk based on the identified disease trees from this study.

Furthermore, our analysis indicated that the most common condition leading to dementia was cerebrovascular disease (either acute or late effect), which supported the findings from several large-scale epidemiological studies that stroke itself can double the risk of dementia and accelerate its onset by 10 years [33,34]. In a United Kingdom–based longitudinal study, the annual incidence of poststroke dementia was 34.3% in patients with severe stroke, 8.2% in patients with minor stroke, and 5.2% in those with transient ischemic attack [35]. This not only supports our findings regarding cerebrovascular diseases (acute or late effect) but also suggests that most late-onset dementia has a mixed etiology. Other than cerebrovascular disease, several cardiovascular conditions, for example, hypertension and coronary atherosclerosis, have been identified in the pathways prior to dementia. Accumulating epidemiological evidence has indicated that cardiovascular diseases and dementia are highly prevalent and that cardiovascular diseases tended to increase the risk of developing dementia [36,37]. Since the brain is a highly vascularized organ and is very susceptible to impaired blood flow and vascular pathology, the heart-brain connection deserves further research attention to construct a systemic approach to protect both major organs. Growing evidence has indicated roles for subclinical carotid atherosclerosis and slow carotid blood flow in cognitive dysfunctions [38], and the disease trees identified in this study strongly support these hypotheses and observations [39,40].

In addition to cardiovascular risk, mood disorders are another major disease entity identified in various disease trees. It has been reported that late-life depression substantially increases the risk of dementia of all types [41,42], and depression is the most consistently reported risk factor for dementia [43]. In fact, not only mood disorders themselves but also the use of psychotropic agents (benzodiazepines, z-hypnotics, antipsychotics, and antidepressants) significantly increased the risk of dementia [11,44]. Older adults with multiple or complex care needs were highly likely to be prescribed psychotropic agents for behavioral symptoms related to cognitive impairment occurring in acute medical or surgical conditions, such as hip fractures. Active acute or multiple chronic conditions tend to worsen the cognitive performance of frail older adults, who need psychotropic agents for treatment or symptom management. Taken together, these factors collectively increased the risk of dementia in older adults. In addition to cardiometabolic risk and neuropsychiatric symptoms, nonspecific infections, *Helicobacter pylori* infections, antacids, or proton pump inhibitors for the gastrointestinal system have been identified as important risk factors for dementia in epidemiological studies [14,45-48].

The abovementioned conditions were well captured in the hierarchical disease triplet clusters we identified, which partly validated the model accuracy of this study. Nevertheless, more clinical and research attention is needed to evaluate identified hierarchical disease triplet clusters to establish the causal relationships of these common diseases or conditions in dementia development in older adults. Meanwhile, the risk of certain common age-related conditions in dementia development may be overlooked. For example, mobility difficulties were merely interpreted as common degenerative musculoskeletal conditions instead of early signs of neurodegeneration. Mobility impairment has been widely recognized as a unique risk factor for dementia of all types, which occurs earlier than memory impairment, such as cognitive frailty, motoric cognitive risk syndrome, or physiocognitive decline syndrome.

Moreover, common nonspecific conditions such as dizziness or vertigo may be suggestive of somatic symptoms of preclinical dementia, and these nonspecific conditions were identified as leaves of the hierarchical disease triplet clusters (disease trees) instead of the roots. By definition, the hierarchical disease triplet clusters captured as the pathways for dementia development in this study had strong sequential relationships such that only conditions meeting the patterns would increase the dementia risk of older adults. Hence, the nonspecific somatic conditions or common degenerative conditions were unlikely to be noise signals in the disease trees and deserve further research attention to clarify the pathoetiologic roles of these nonspecific conditions.

Another merit of this study is the clinical implications of mood disorders identified in the hierarchical disease triplet clusters. Our previous pharmacoepidemiologic studies indicated that the use of a higher defined daily dose or combination of different categories of psychotropic agents significantly increased the risk of incident dementia [11], even though not every patient had major psychiatric conditions. The commonly seen nonspecific psychiatric conditions, particularly mood disorders, identified in the hierarchical disease triplet clusters should automatically alert clinicians to the potential risk of dementia. Once again, the machine learning methods enabled this study to validate the clinical significance of these nonspecific conditions in dementia development that should be implemented in electronic health records to capture at-risk populations for multidomain interventions.

### Limitations

Despite all efforts made in this study, there are still some limitations. First, the machine learning methods used in this study clearly identified statistically significant disease trees to predict incident dementia, but it remained difficult to explain all findings. However, our results are strongly compatible with several potential pathological mechanisms of dementia that validated the findings of this study in the hypothetical context of dementia development. Second, we need an external data set to examine whether model overfitting appeared in the machine learning model. Since the NHIRD is a nationwide setting, appropriate data sets from other countries are needed for external validation. Third, in our study, we focused solely on identifying disease and symptom clusters and sequences prior to dementia to predict the risk of dementia. We did not include other crucial risk factors and variables, such as neuroimaging, clinical features, cognitive function, genetic data, and behavioral variables. Although we recognize that incorporating these data may enhance the accuracy of our dementia risk prediction, it may not be practical to include all of these factors in routine clinical practice. Thus, we aimed to use only disease and symptom clusters and sequences prior to dementia, which can be easily accessed from medical records, to identify dementia risk and have the potential for clinical application in dementia prevention. Fourth, we did not consider the time interval between diseases or symptoms in our hierarchical cluster analysis. Although incorporating time between diagnoses could potentially offer additional clinical insights, our study already identified a large number of features using hierarchical cluster analysis in the by-group LASSO and 1-model LASSO methods, with up to 2513 and 181 different features, respectively. Including the time interval between diagnoses in the model would further increase its complexity and make it difficult to reduce the dimensionality of the model for identifying clinically meaningful disease pathways. However, future studies incorporating time intervals into the disease pathway, based on the findings of our study, are warranted. Last but not least, we were unable to access data that are not routinely collected in a claims database, such as smoking, alcohol drinking, exercise, and other lifestyle factors. However, this is the universal challenge for all studies, especially those using claims data.

### Conclusions

The development of dementia using real-world data is more complex than epidemiological studies such that the joint effects of leading roles (cardio- or cerebrovascular disease) and supporting roles (mental conditions, locomotion difficulties, infections, and nonspecific neurological conditions) provide a more comprehensive story of dementia development. Additional studies using data from other countries are needed for external validation, and more comprehensive strategies, for example, multidomain intervention or integrated care for multimorbidity, are needed to prevent dementia or to improve the quality of care.

## Appendix

Multimedia Appendix 1 Forty-eight disease trees with distinct roots in the final model.

## Abbreviations

NHIRD: National Health Insurance Research Database
SVM: support vector machine
LASSO: least absolute shrinkage and selection operator
WHO: World Health Organization

## Data Availability

Due to privacy issues of data and the data protection policy from the Health and Welfare Data Science Center, Ministry of Health and Welfare, Taiwan, it is not available to the community via open repository.
