# Supplementary material for: Using Hypothesis-Led Machine Learning and Hierarchical Cluster Analysis to Identify Disease Pathways Prior to Dementia: Longitudinal Cohort Study
Source: J Med Internet Res. 2023 Jul 26;25:e41858. doi: 10.2196/41858 (PMC10413246; doi:10.2196/41858)

**Figure S1.** 48 disease trees with distinct roots in the final model

**Figure S1-1**


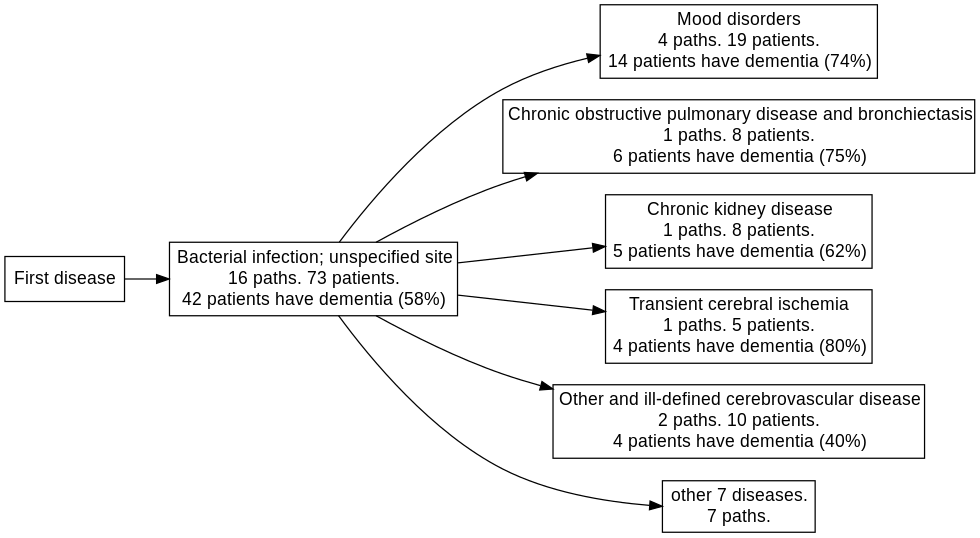


**Figure S1-2**


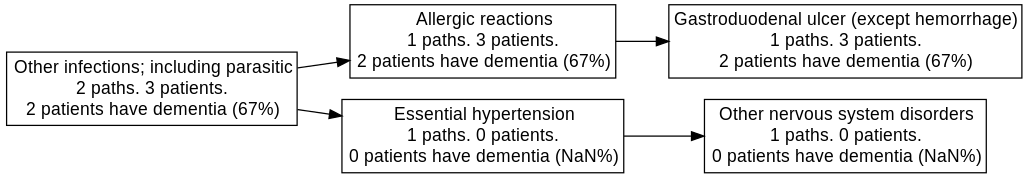


**Figure S1-3**


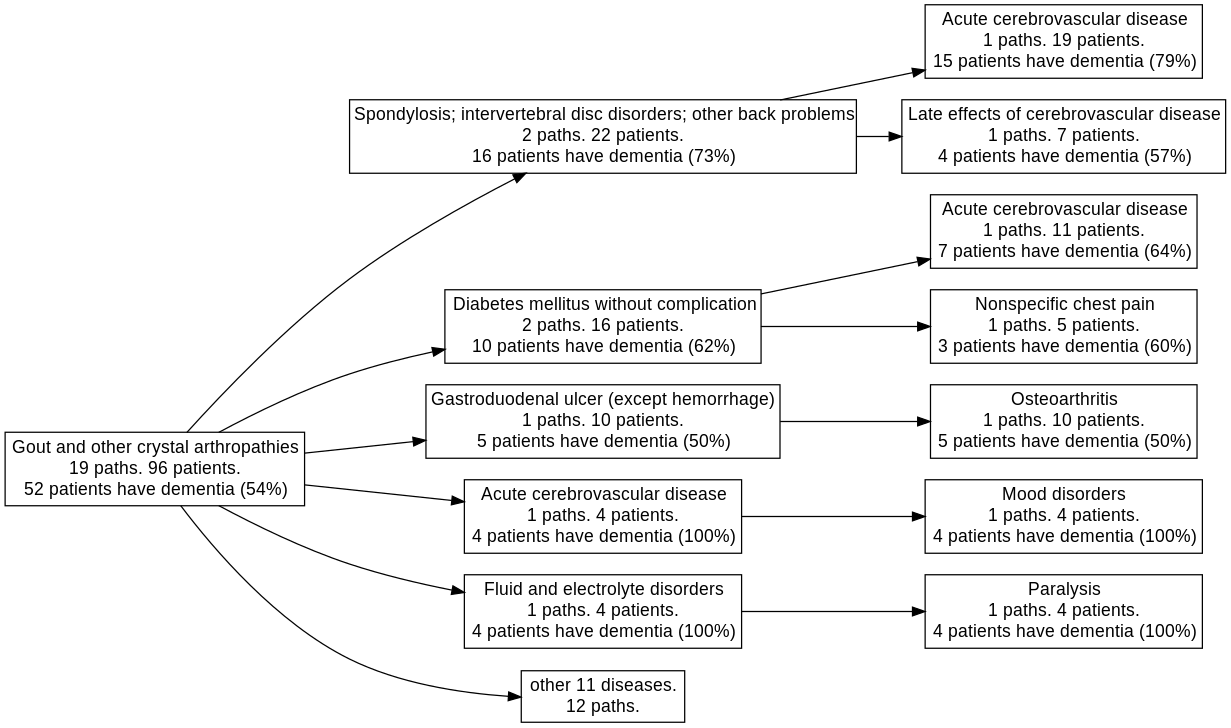


**Figure S1-4**


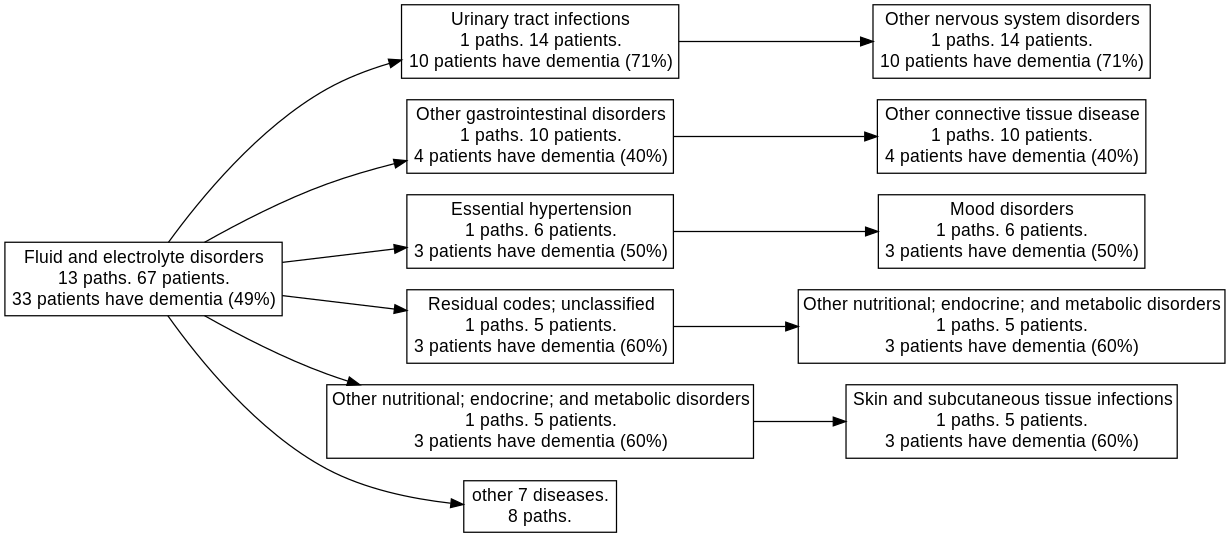


**Figure S1-5**


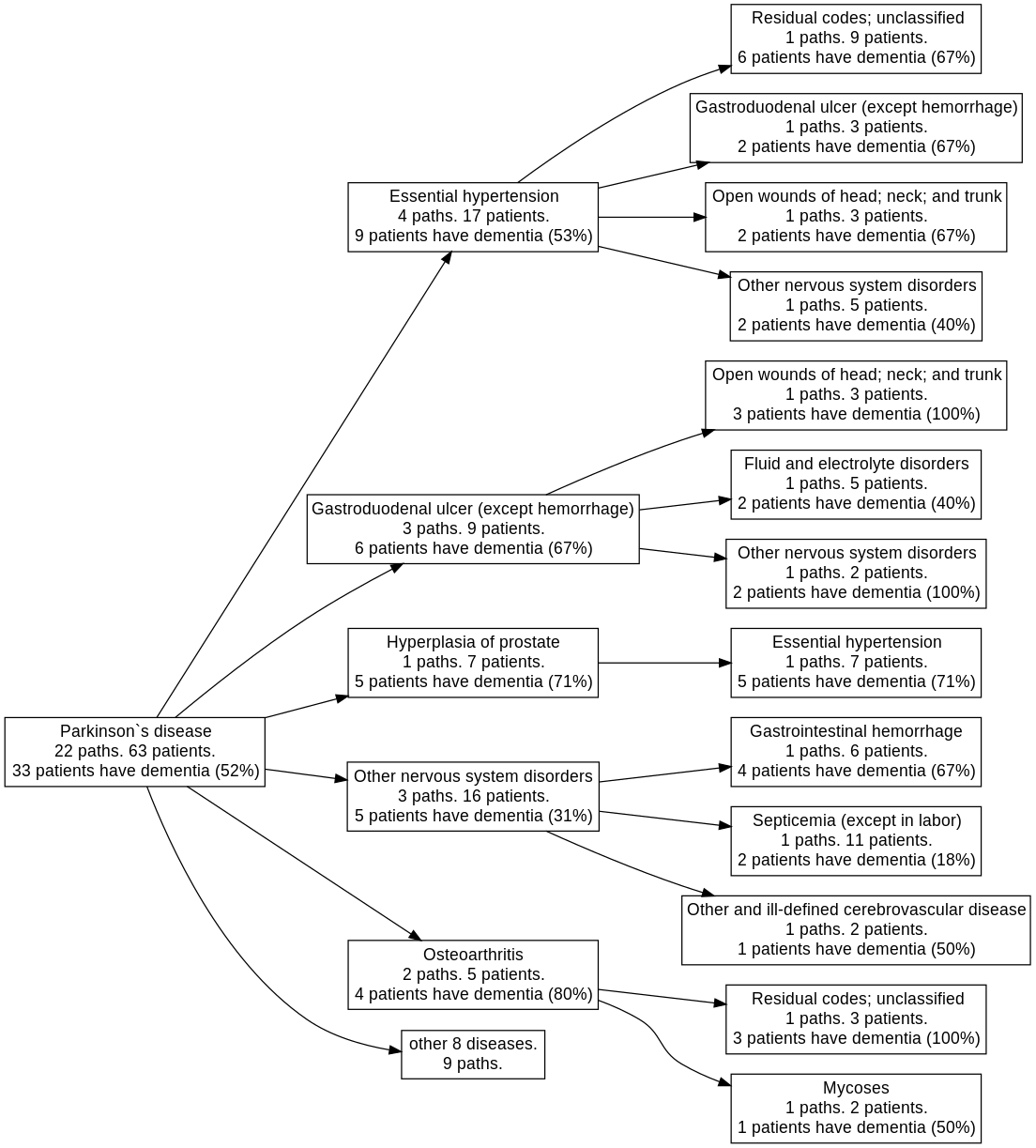


**Figure S1-6**


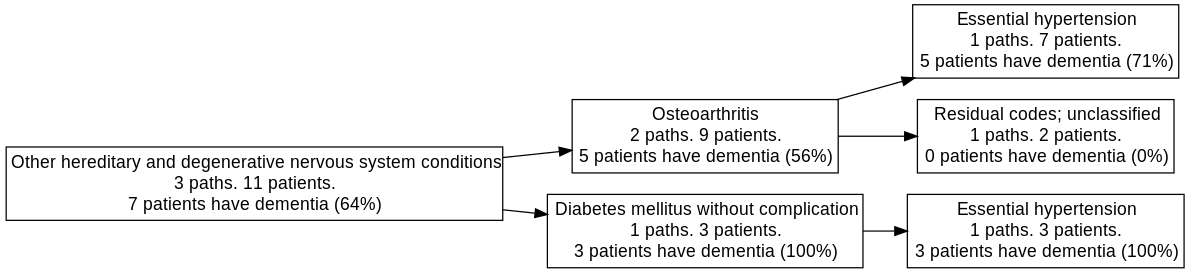


**Figure S1-7**


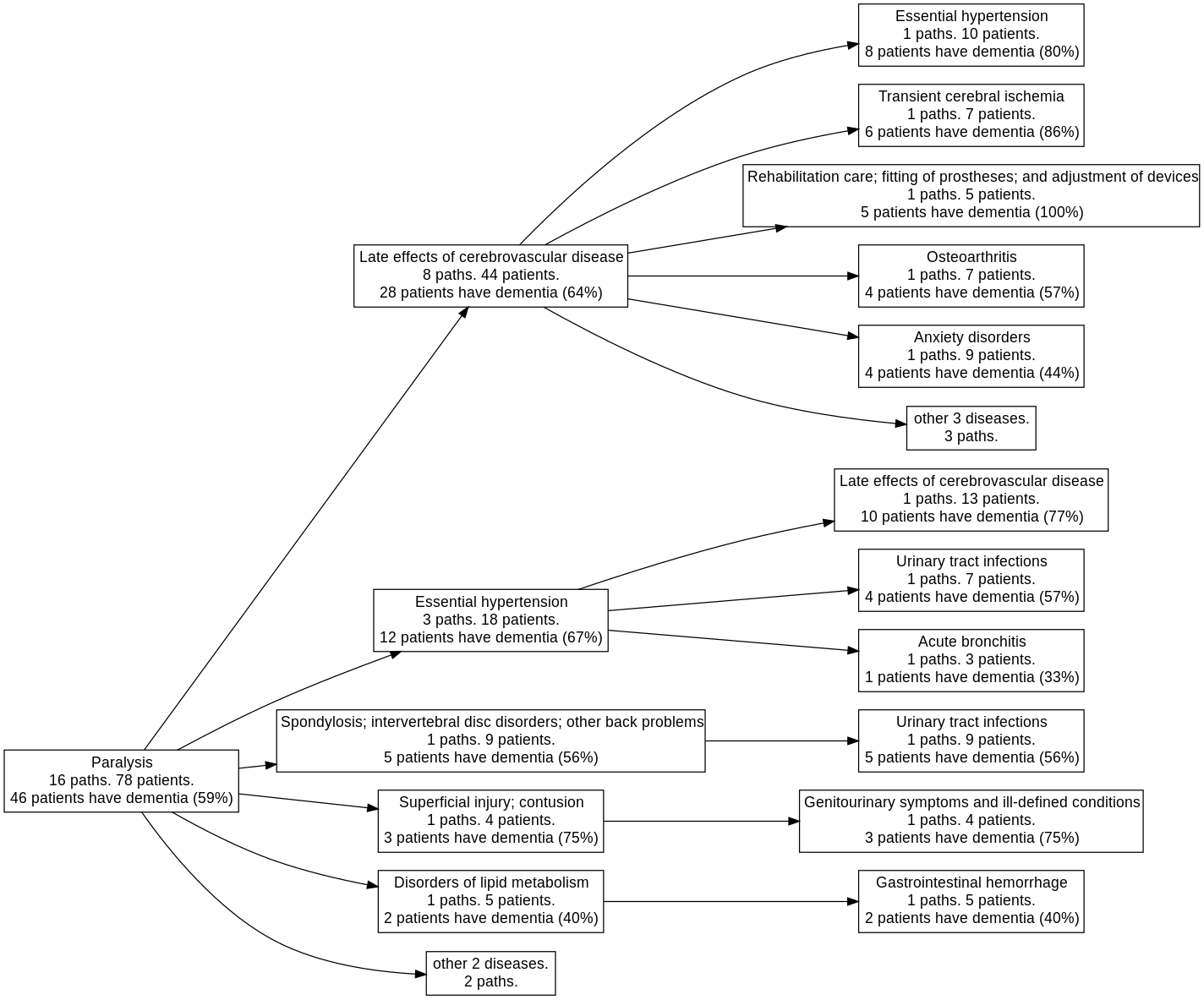


**Figure S1-8**


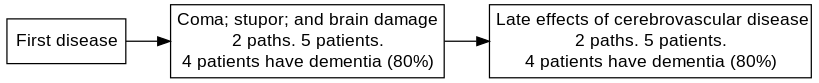


**Figure S1-9**


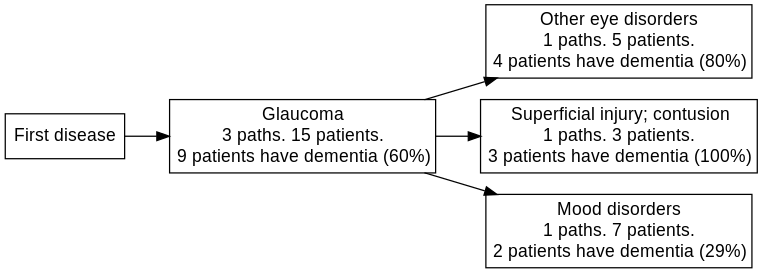


**Figure S1-10**


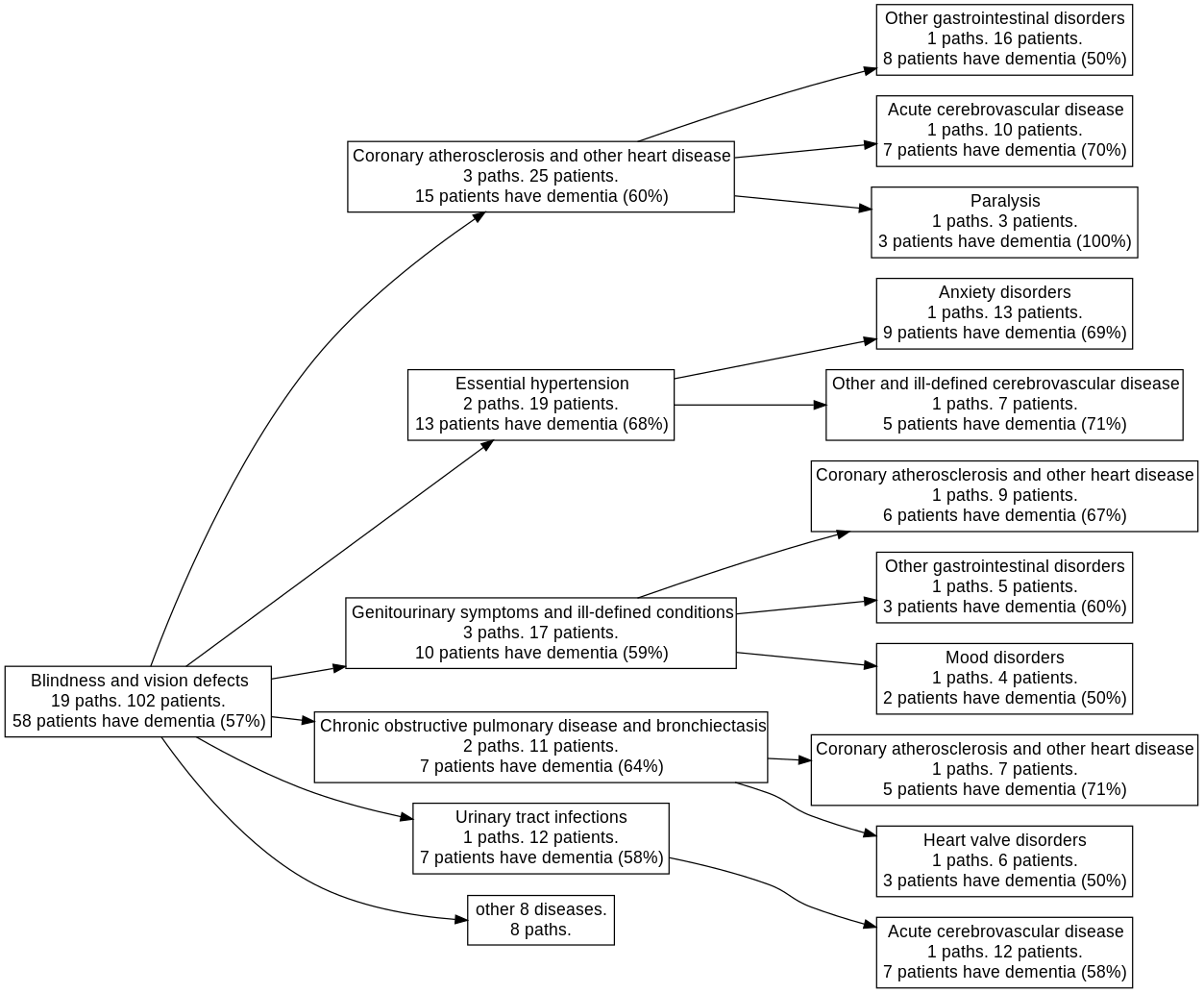


**Figure S1-11**


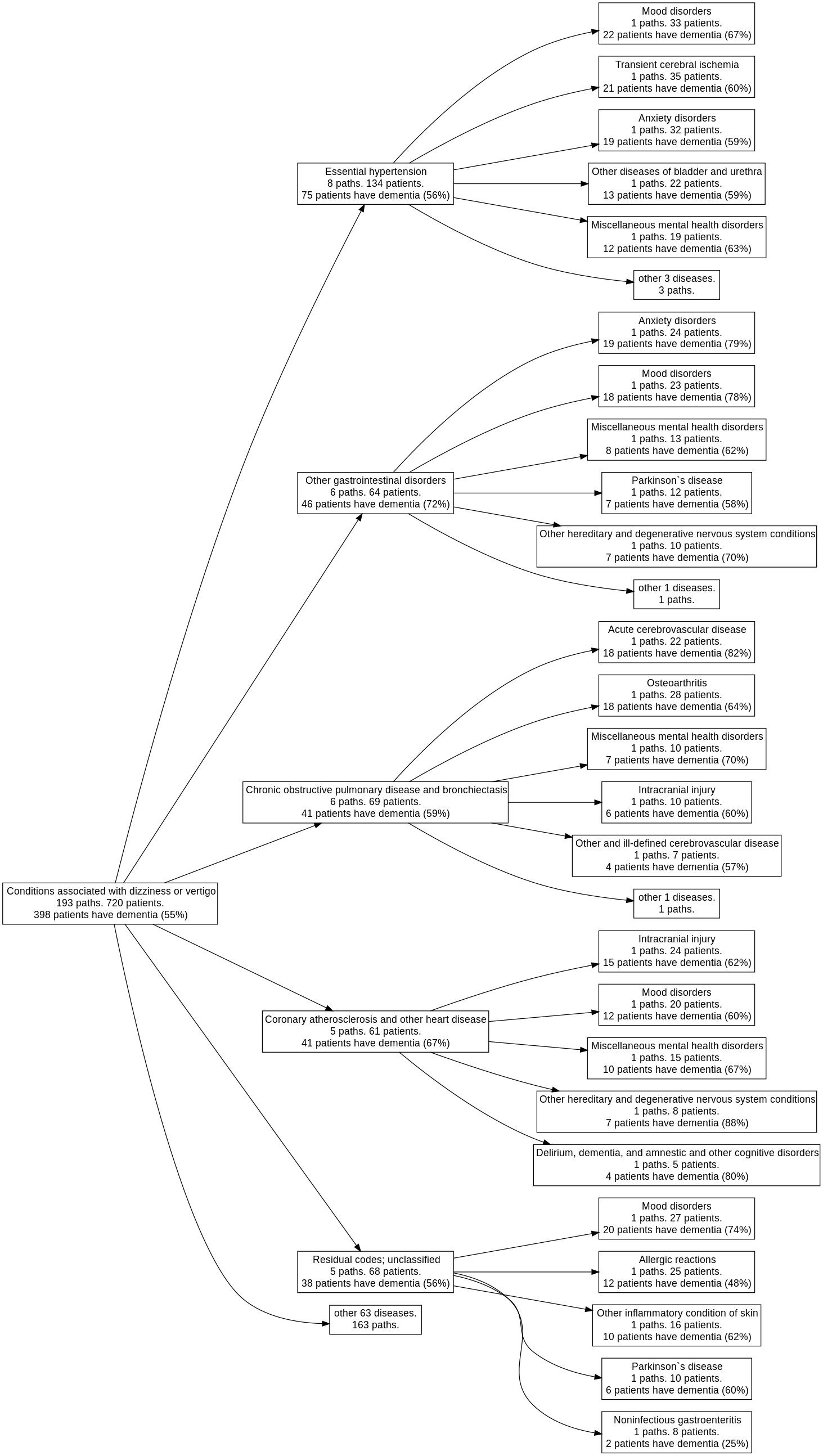


**Figure S1-12**


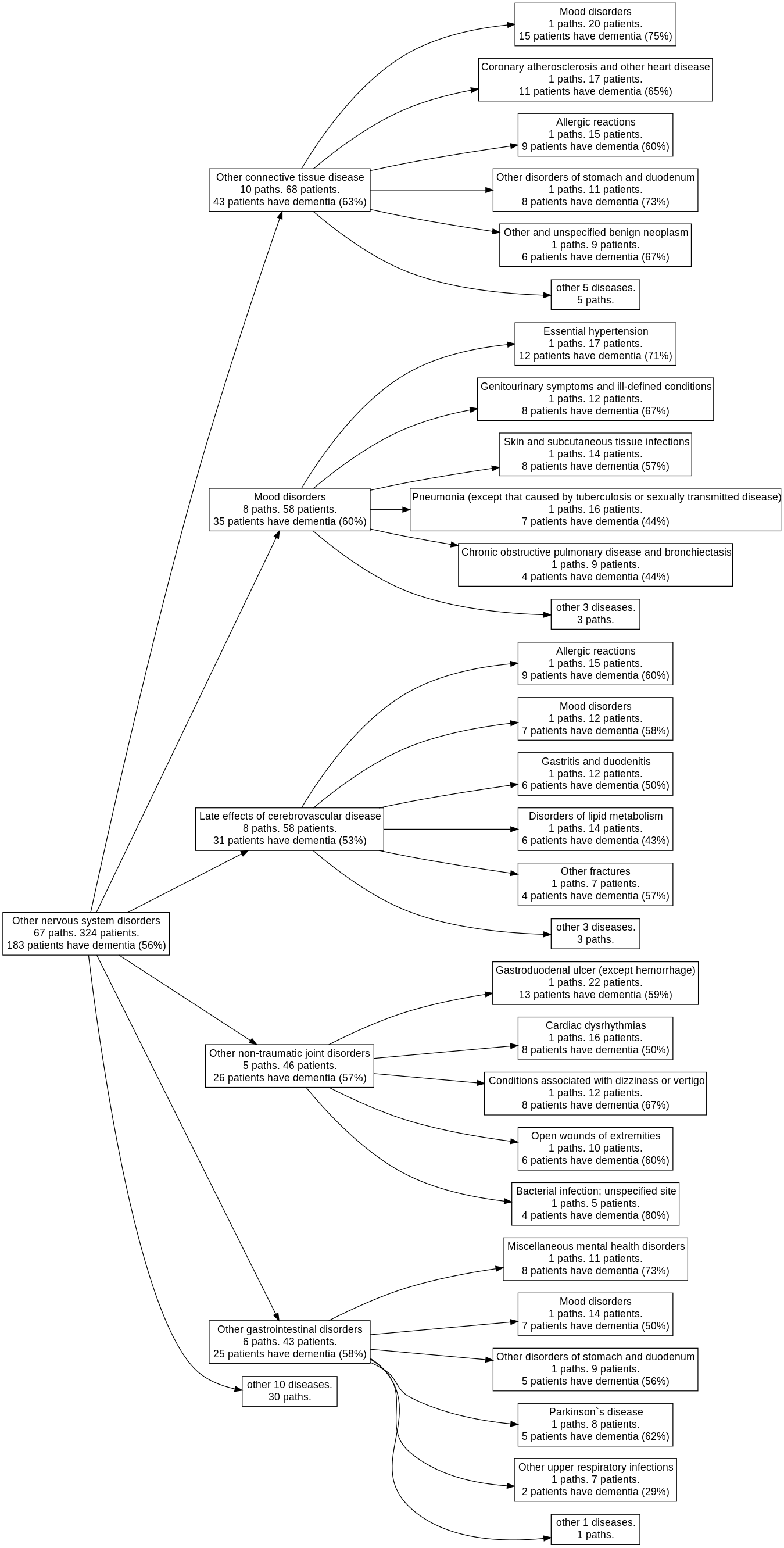


**Figure S1-13**


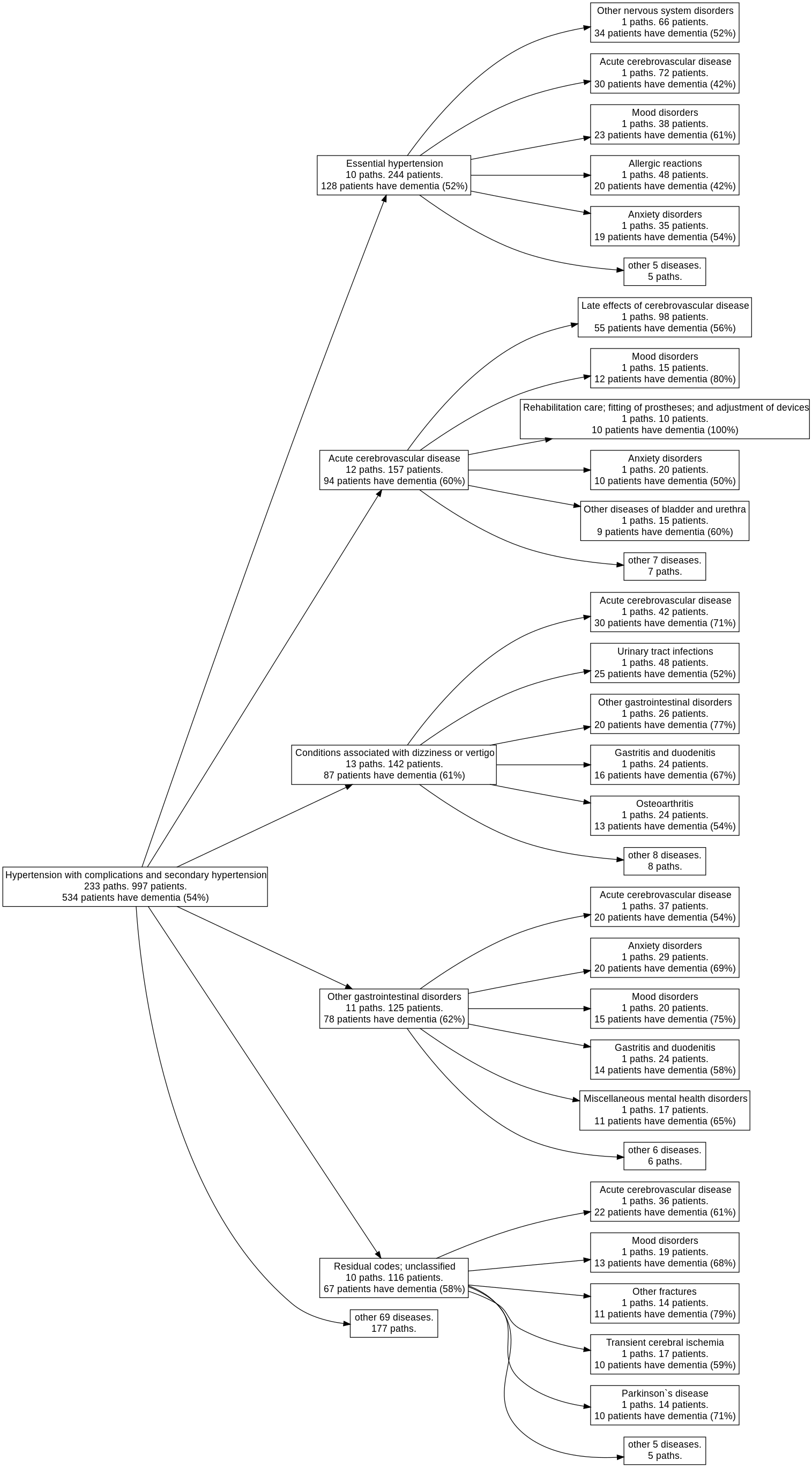


**Figure S1-14**


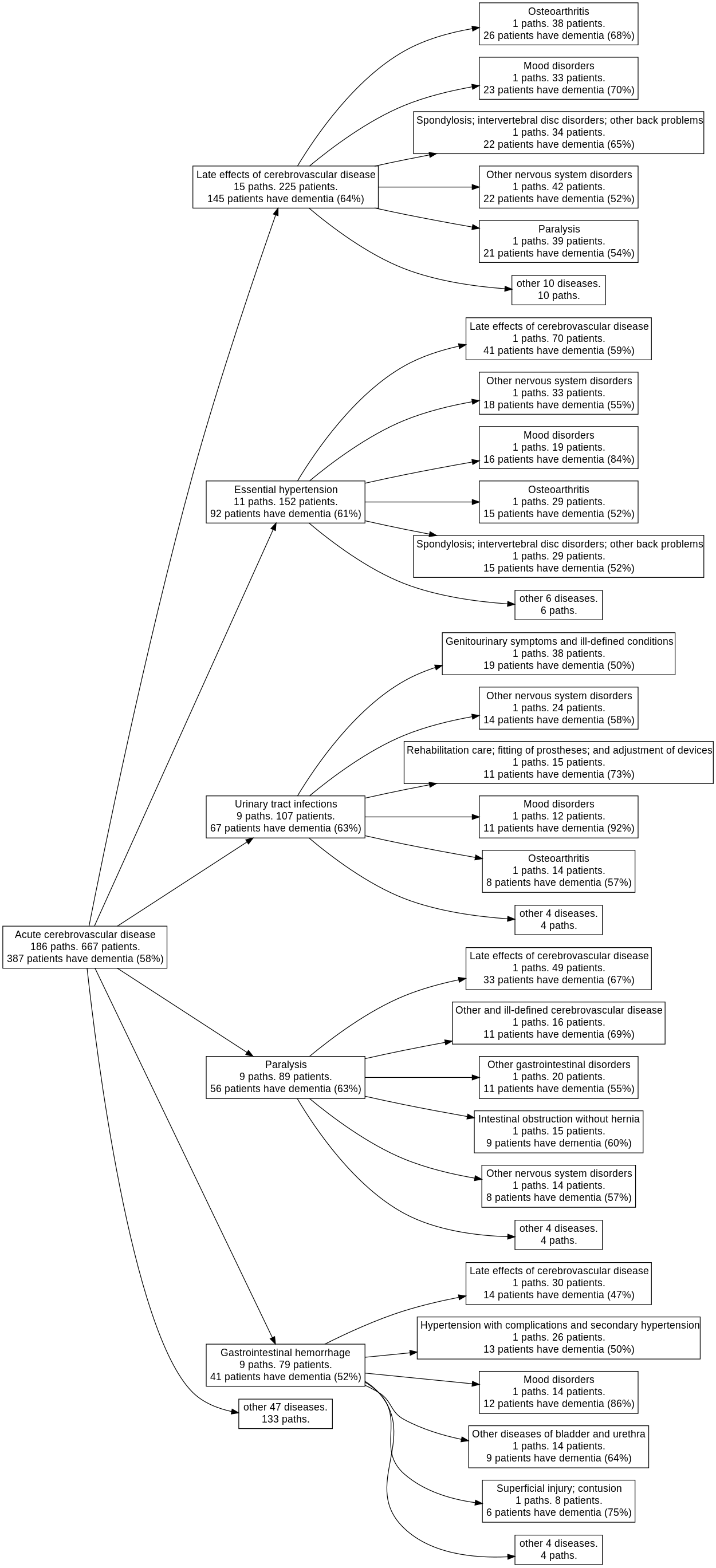


**Figure S1-15**


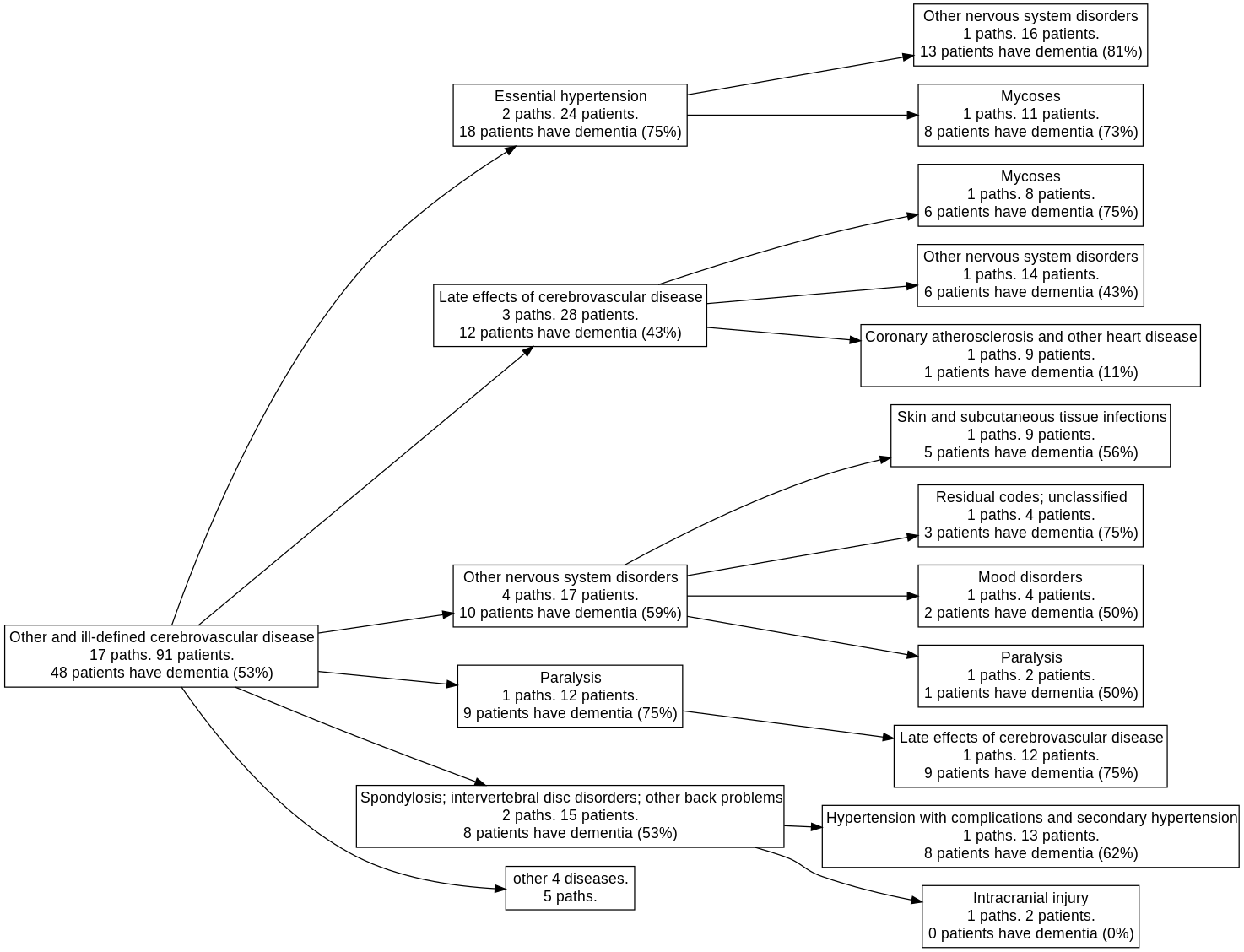


**Figure S1-16**


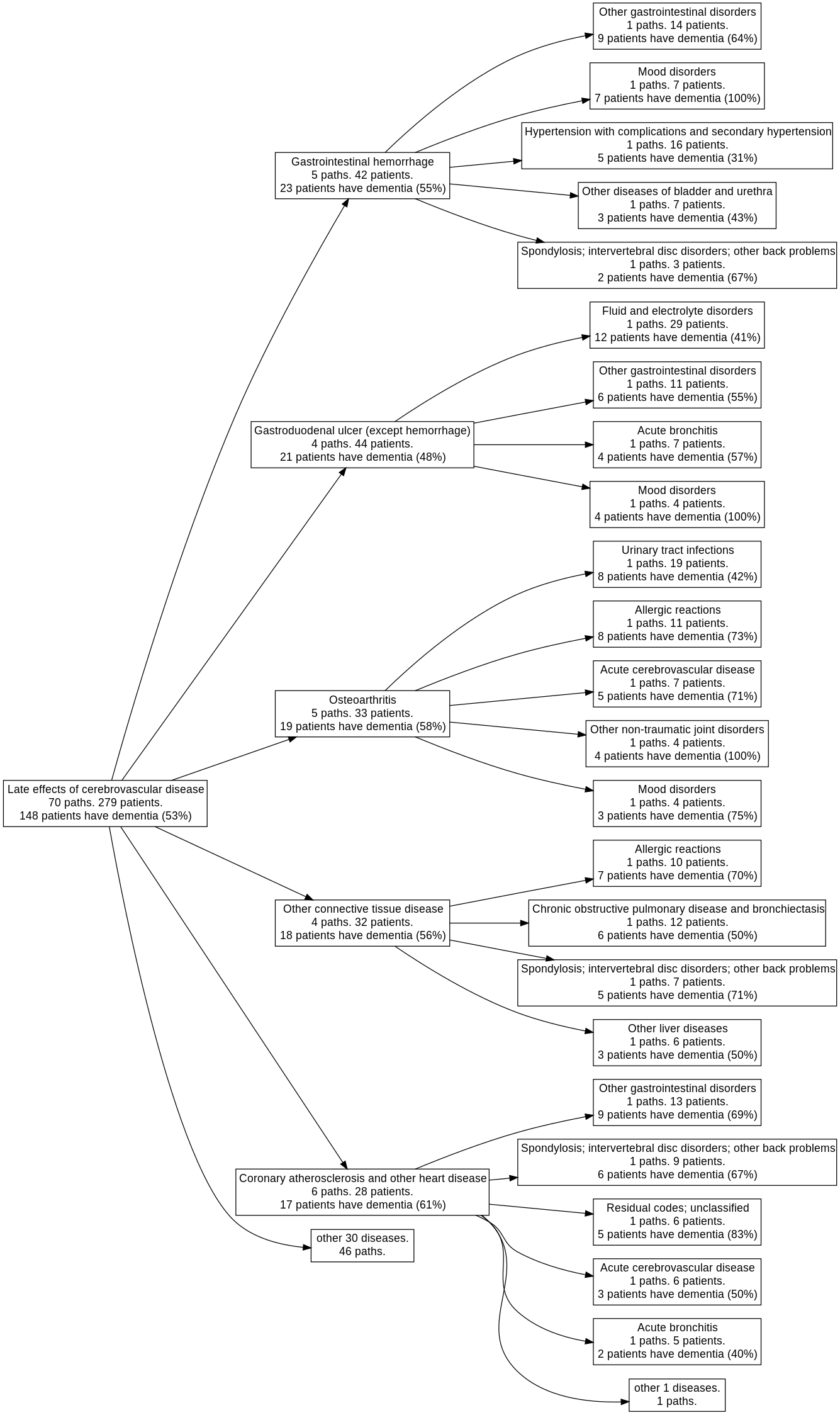


**Figure S1-17**


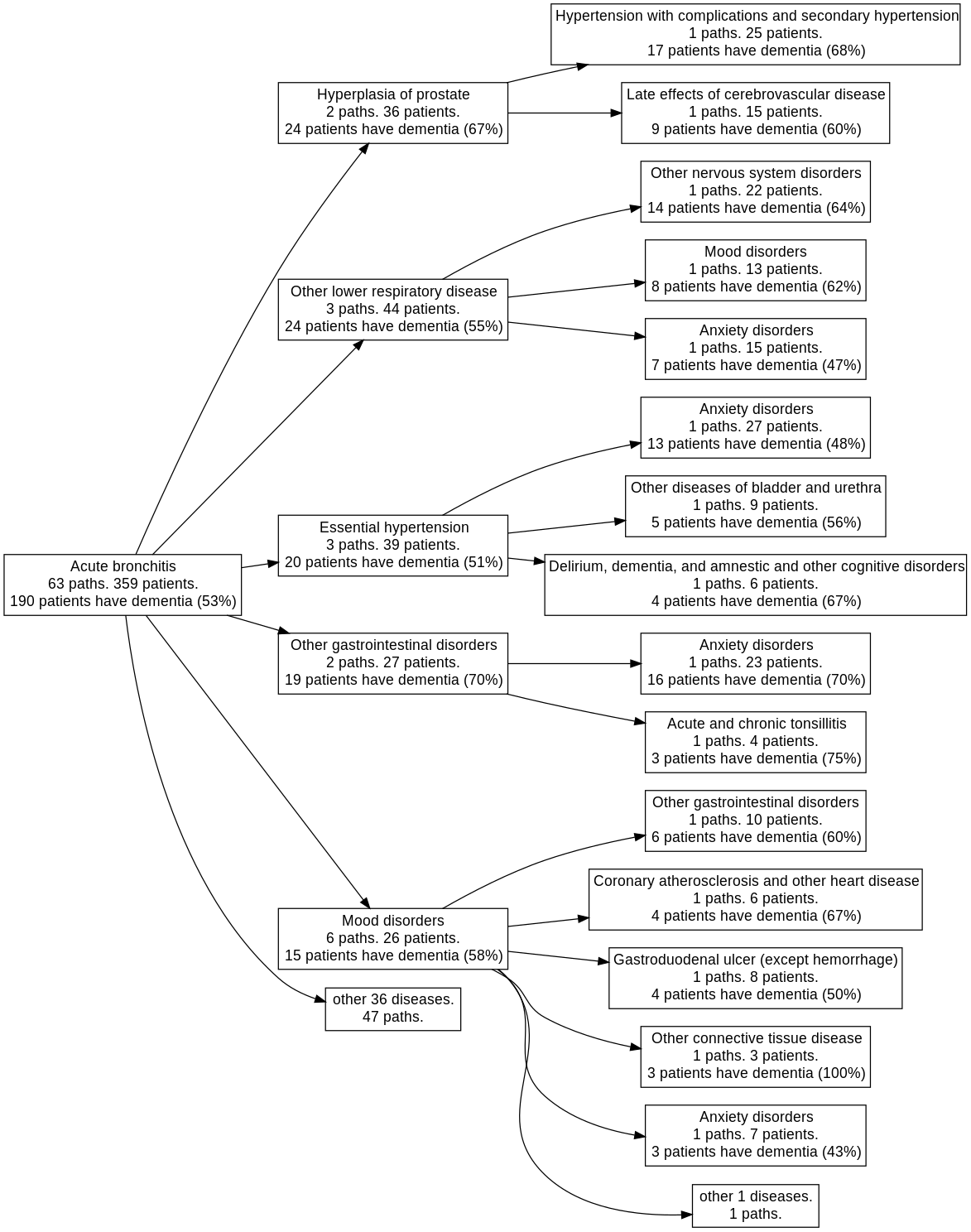


**Figure S1-18**


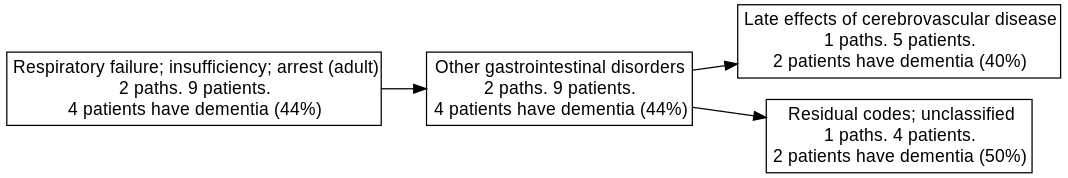


**Figure S1-19**


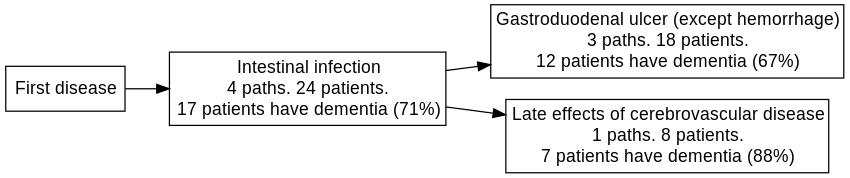


**Figure S1-20**


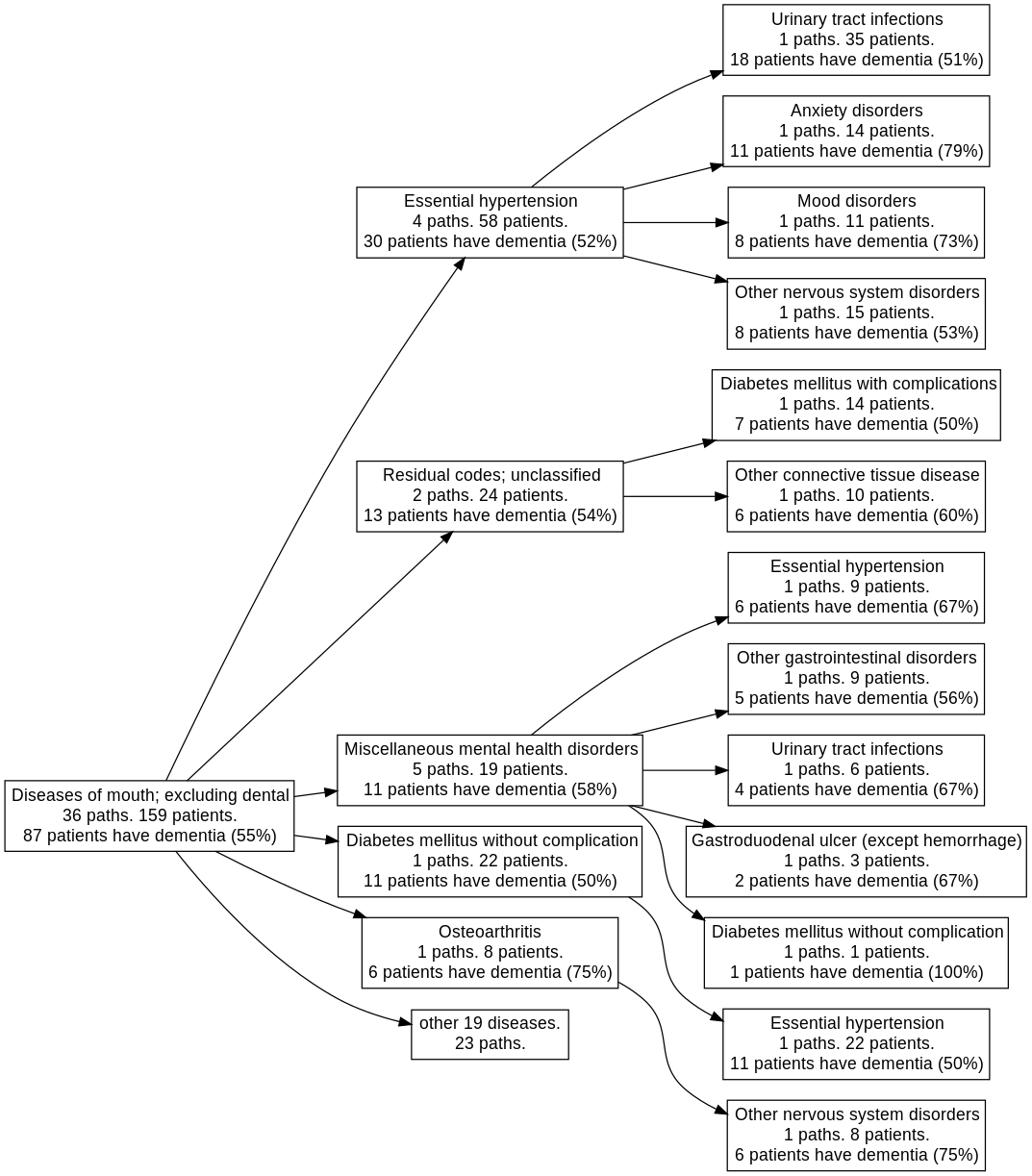


**Figure S1-21**


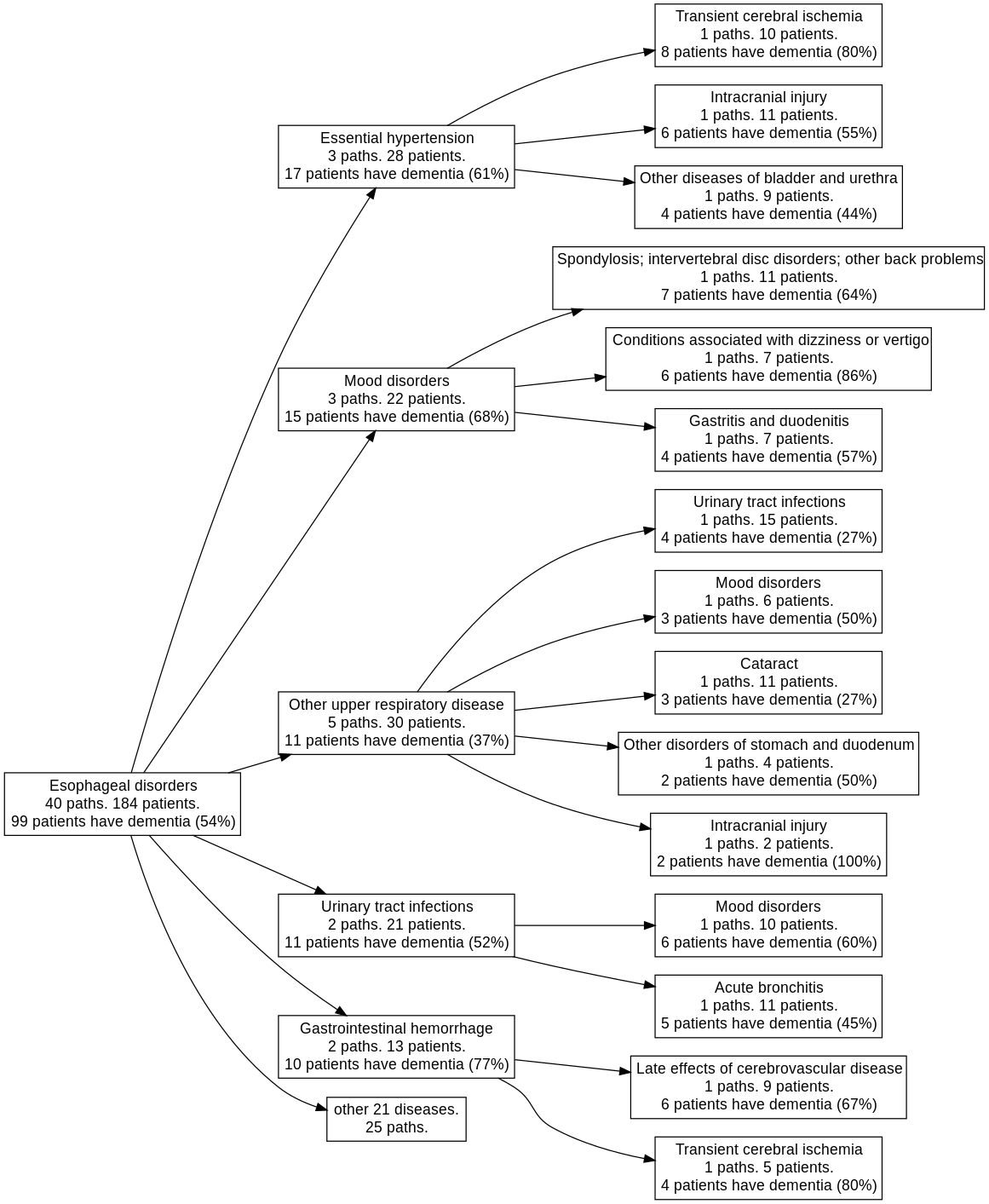


**Figure S1-22**


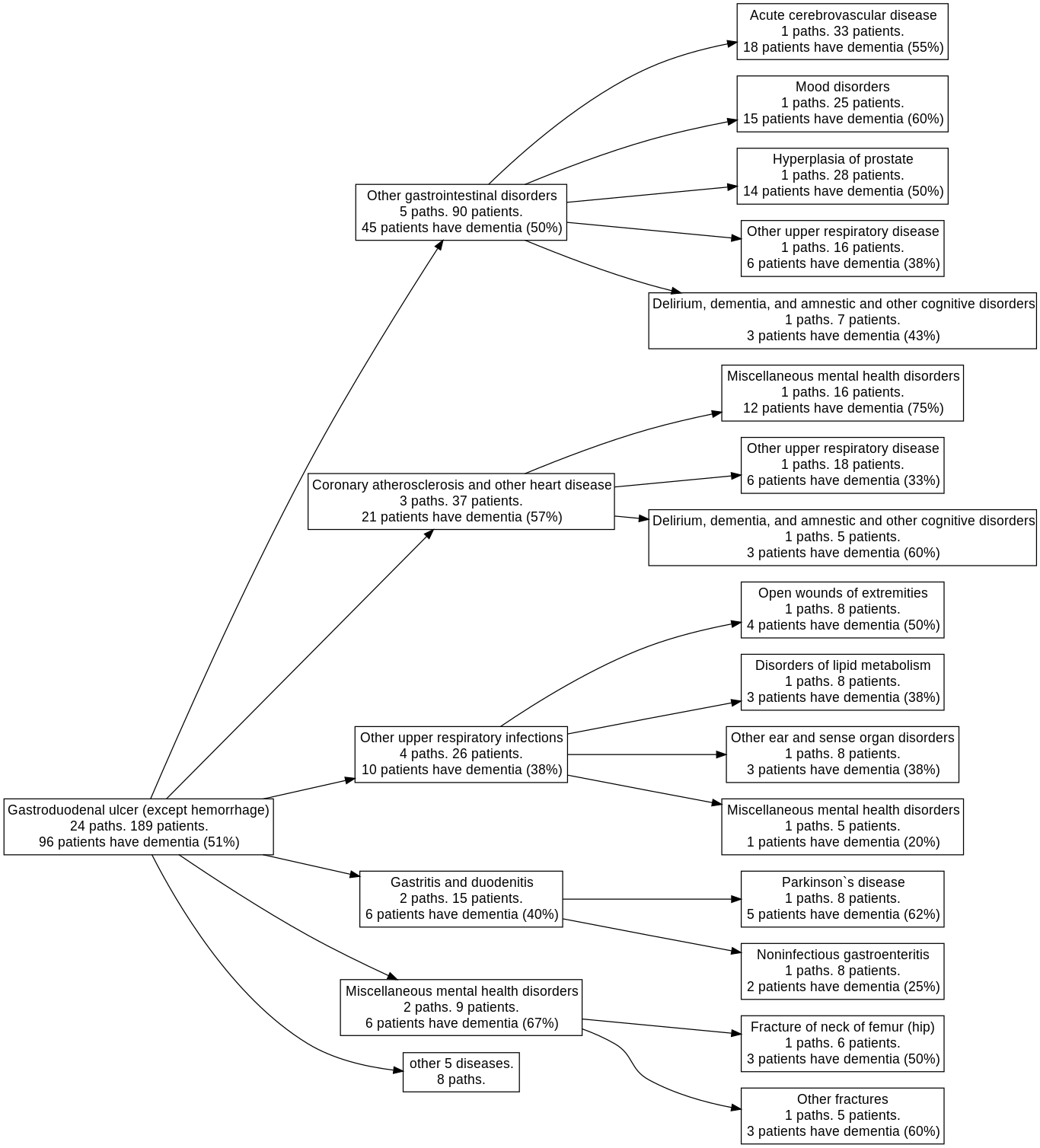


**Figure S1-23**


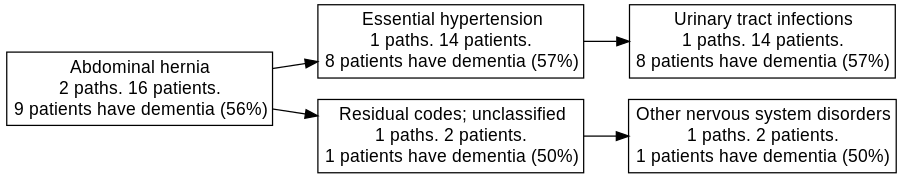


**Figure S1-24**


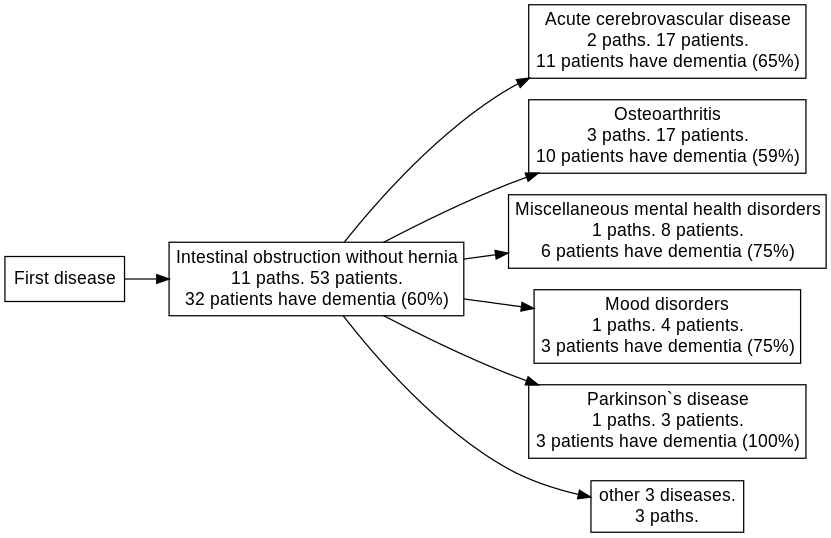


**Figure S1-25**


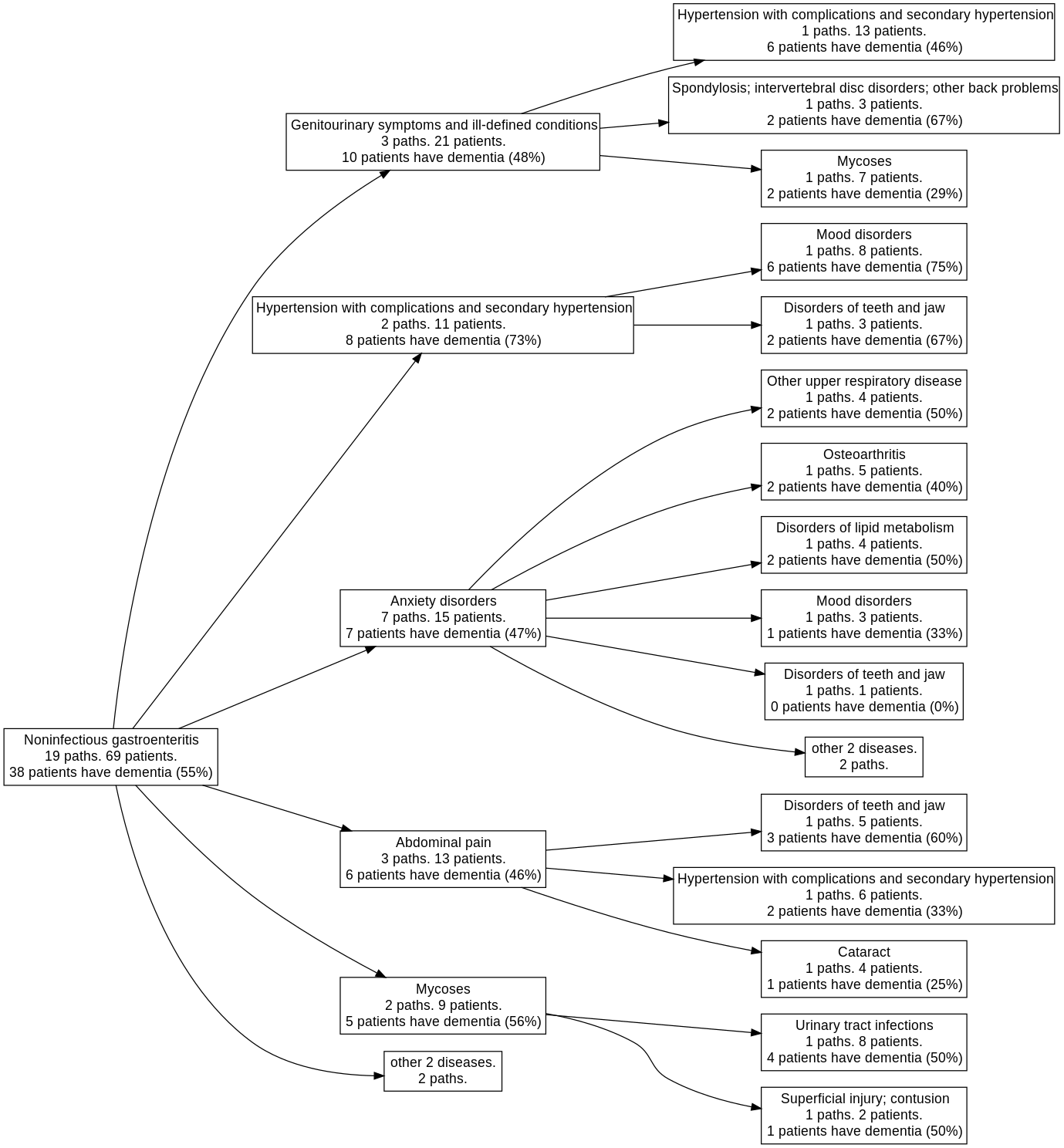


**Figure S1-26**


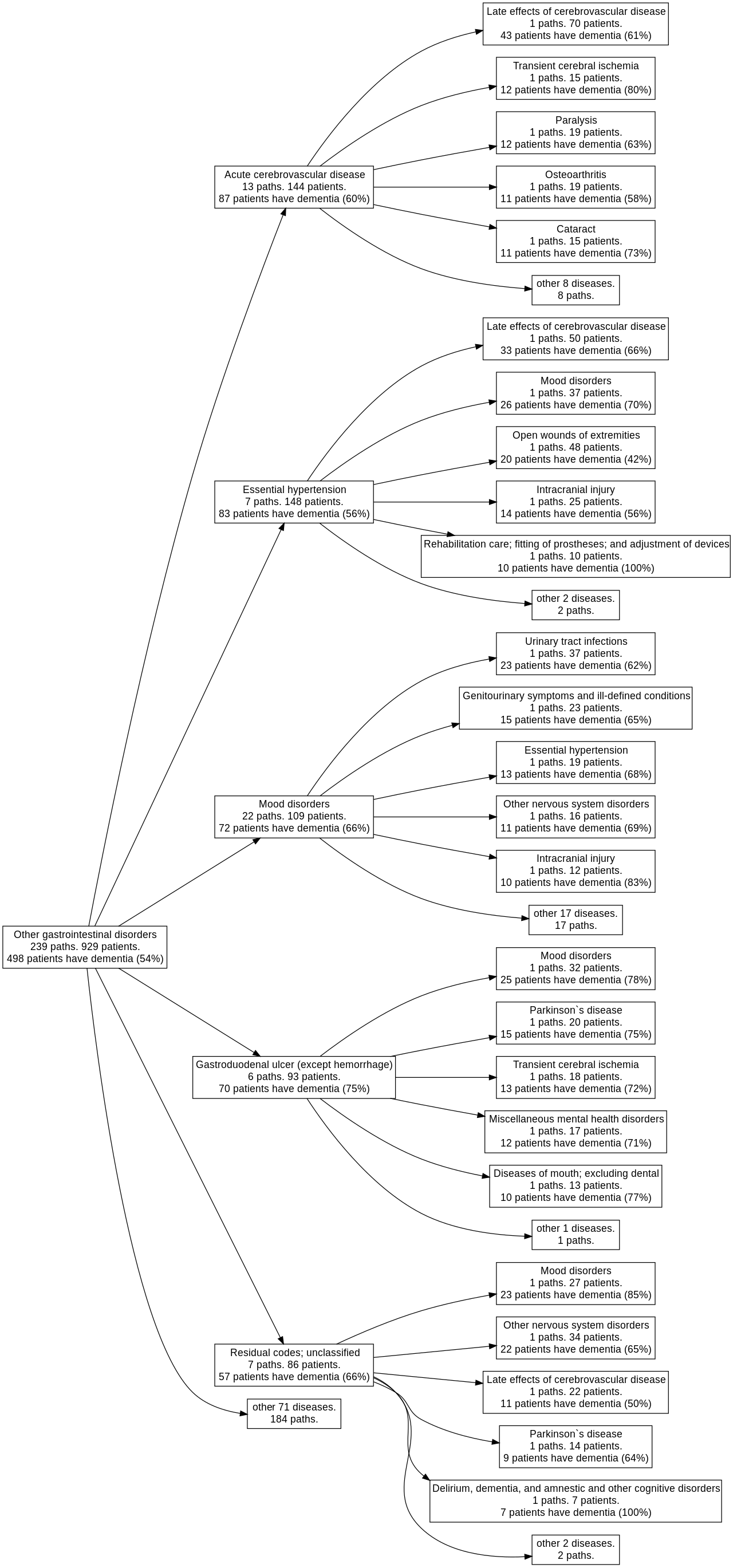


**Figure S1-27**


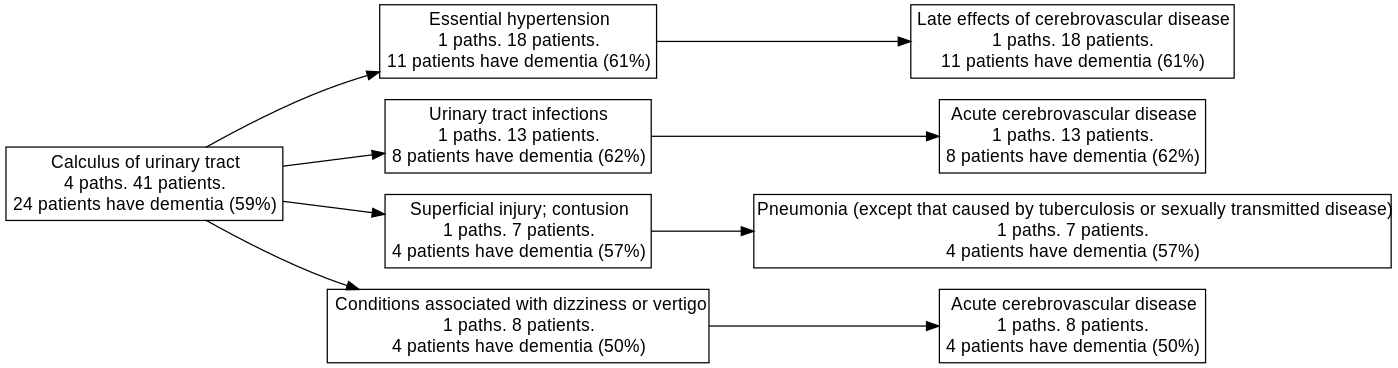


**Figure S1-28**


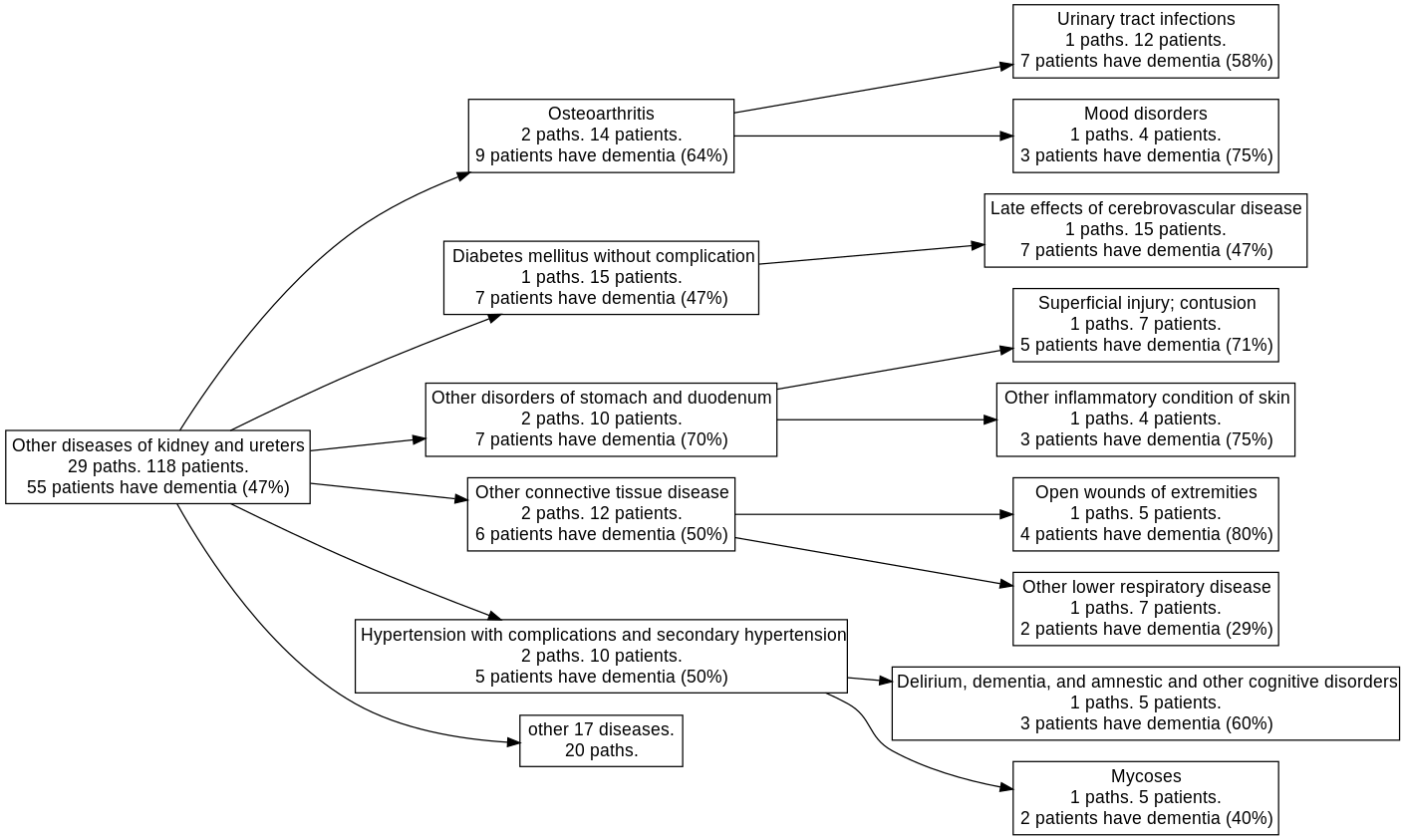


**Figure S1-29**


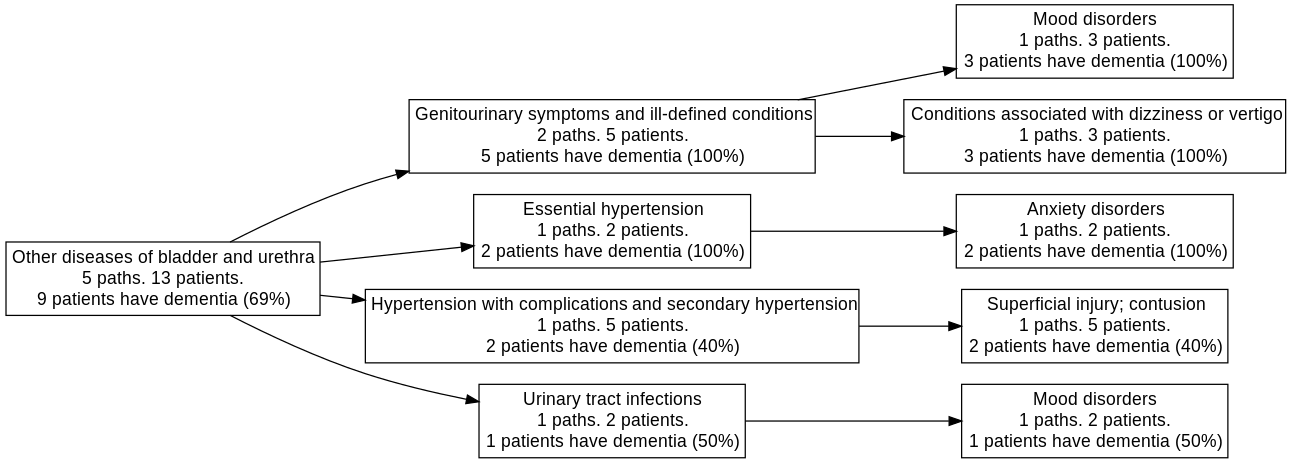


**Figure S1-30**


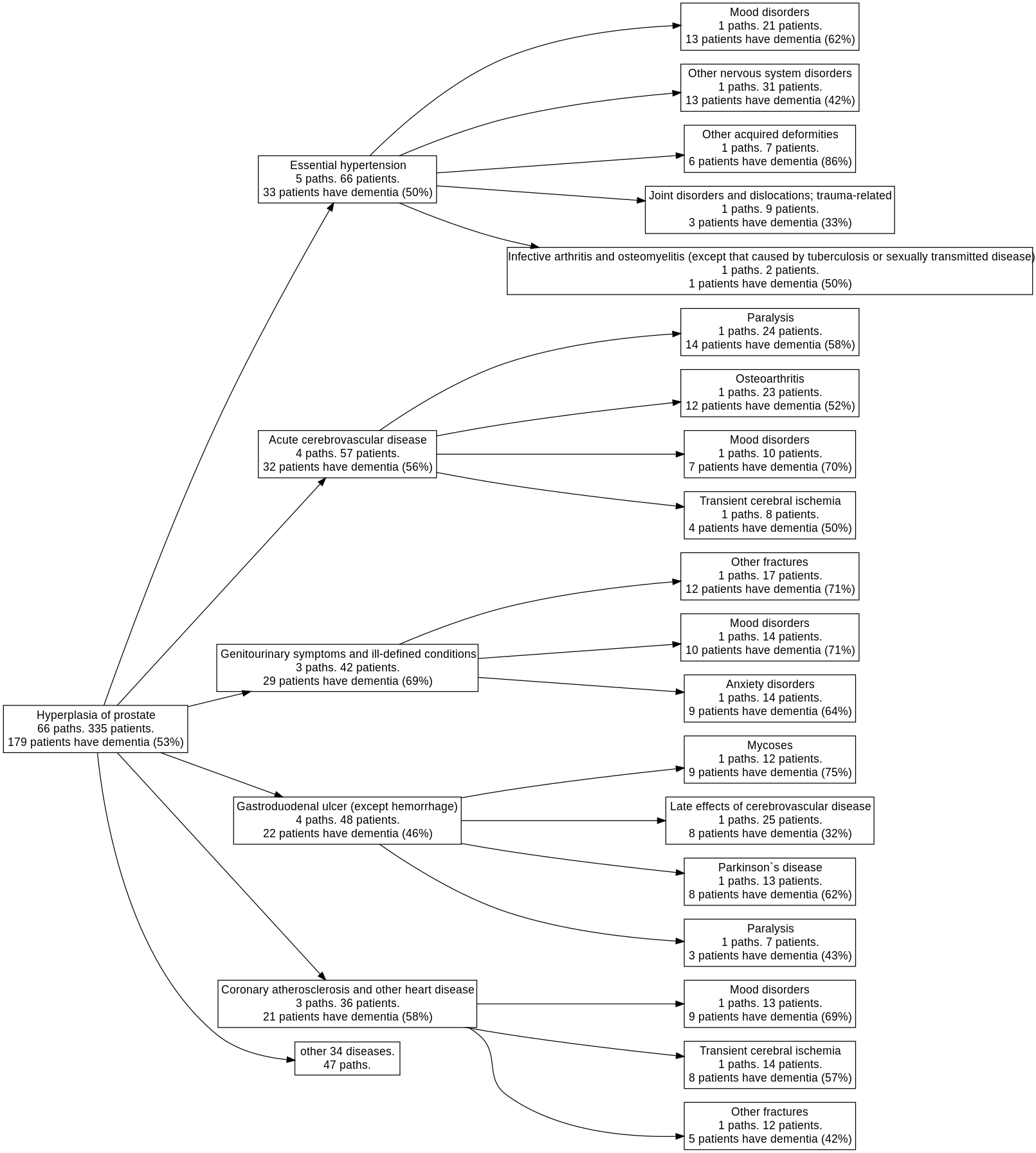


**Figure S1-31**


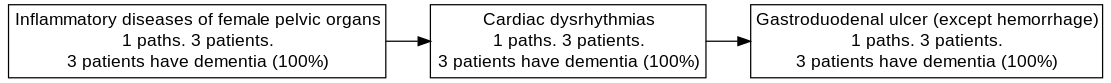


**Figure S1-32**


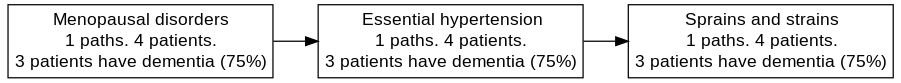


**Figure S1-33**


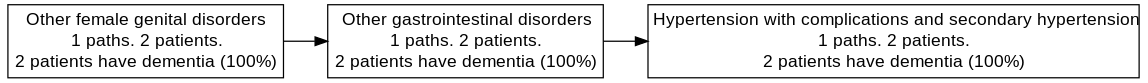


**Figure S1-34**


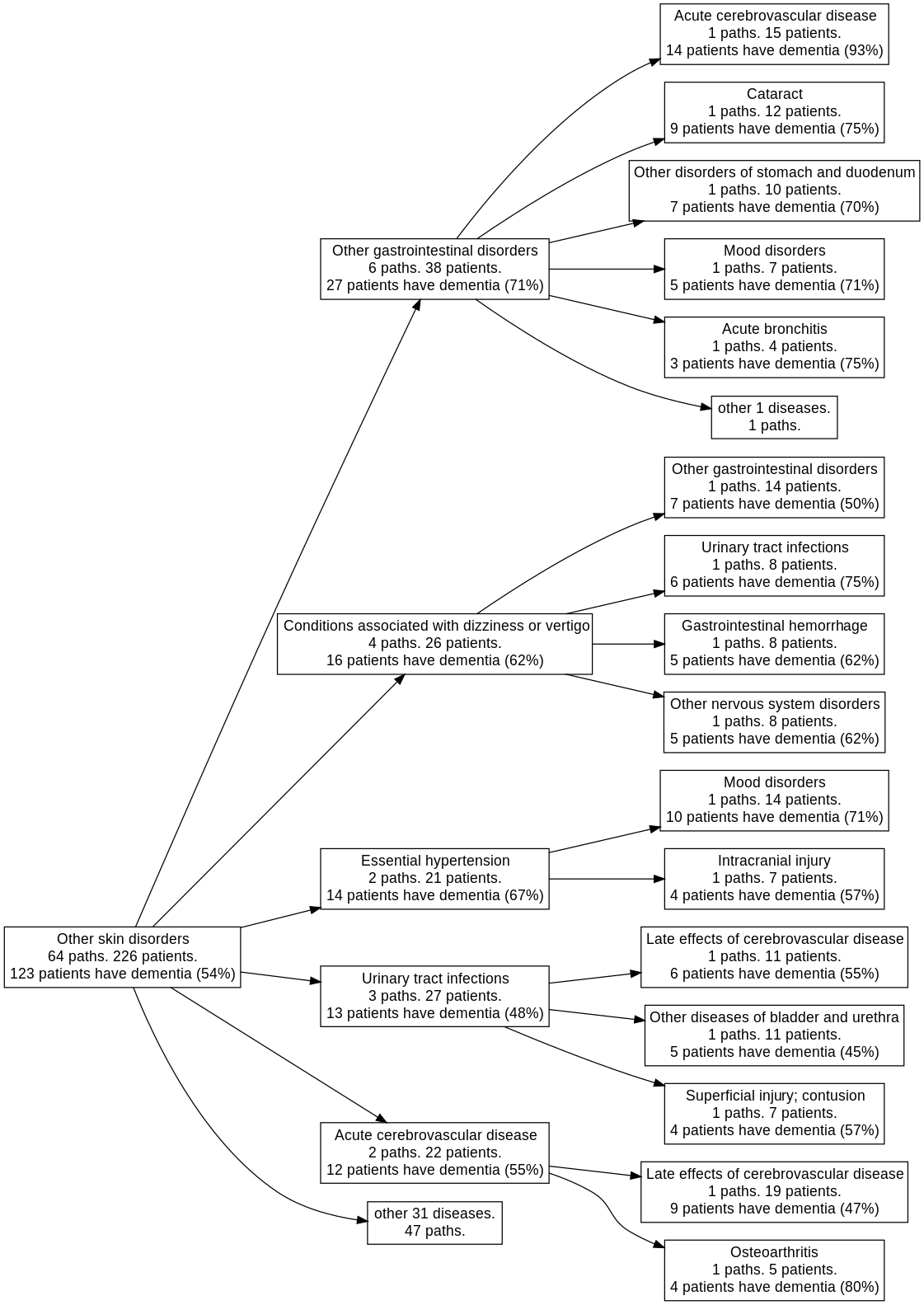


**Figure S1-35**


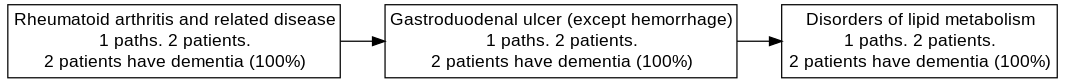


**Figure S1-36**


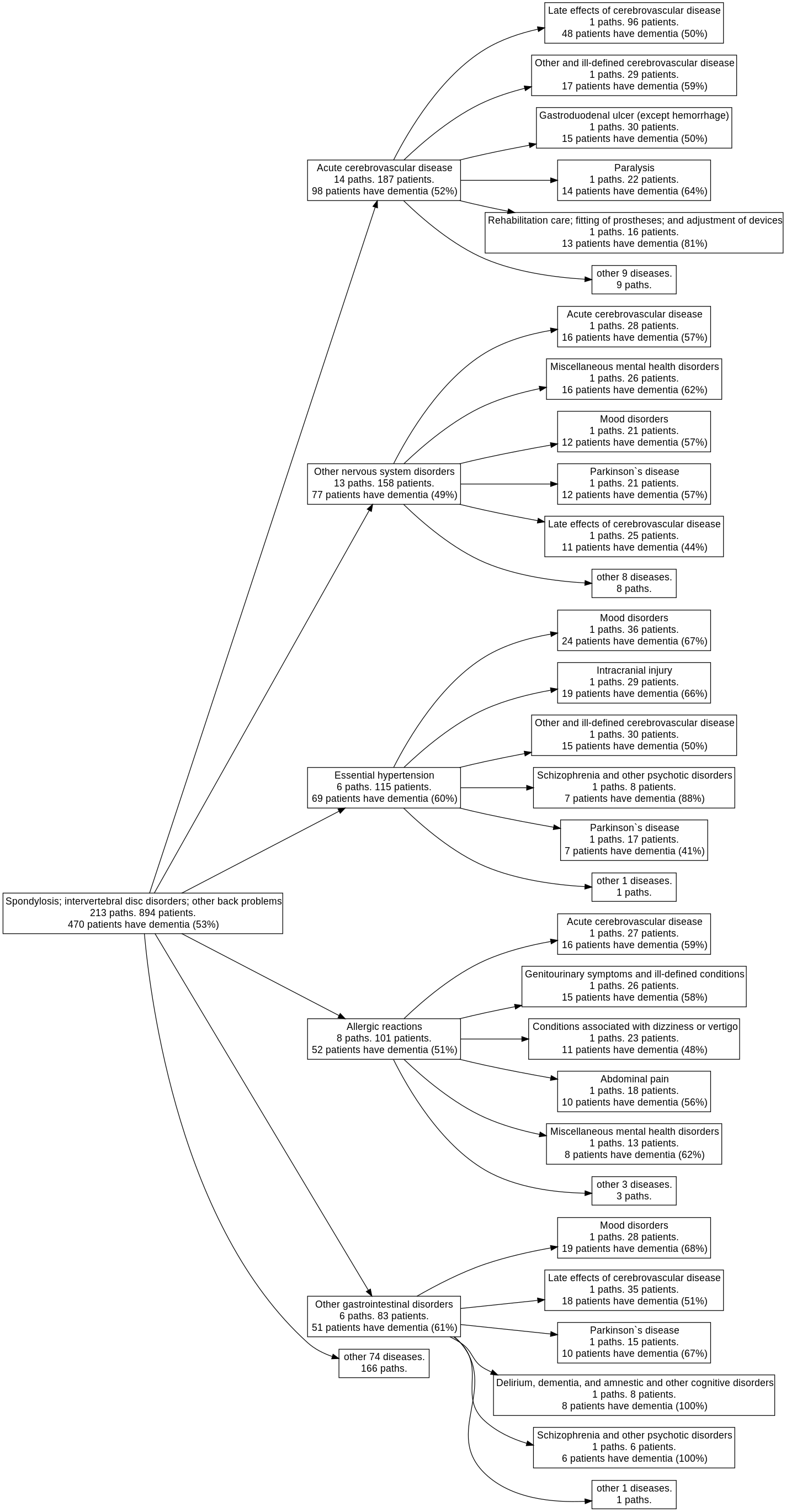


**Figure S1-37**


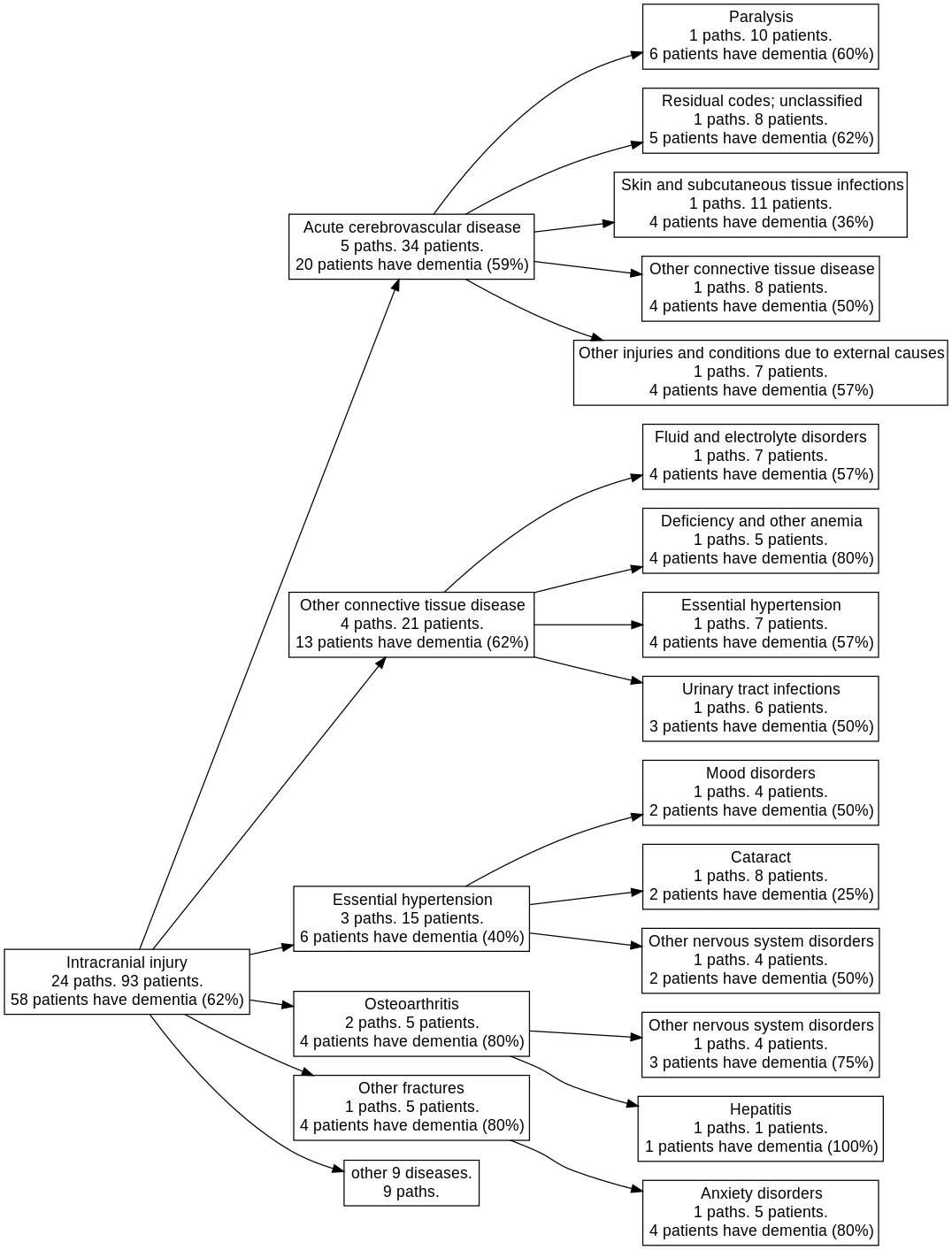


**Figure S1-38**


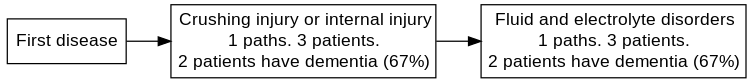


**Figure S1-39**


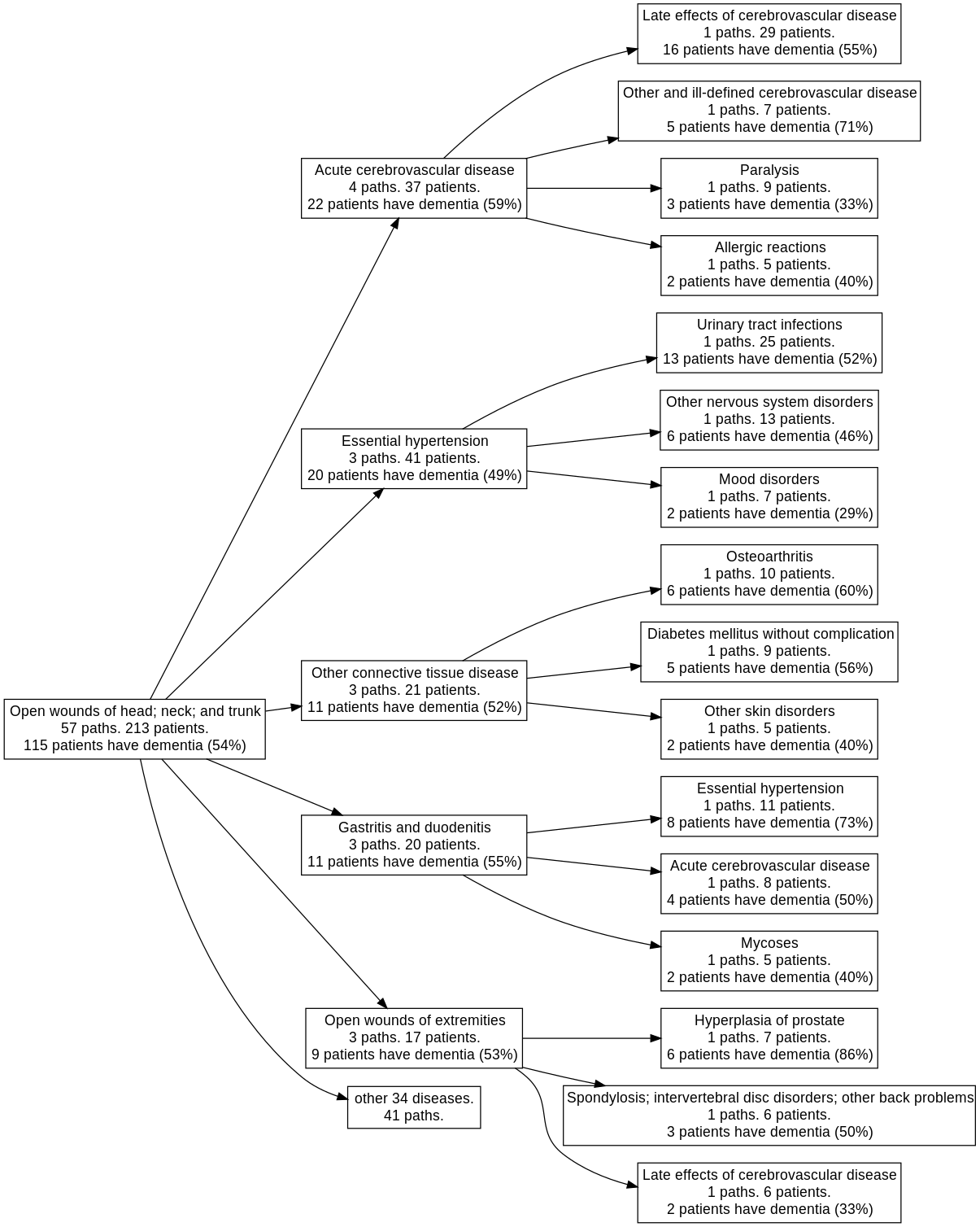


**Figure S1-40**


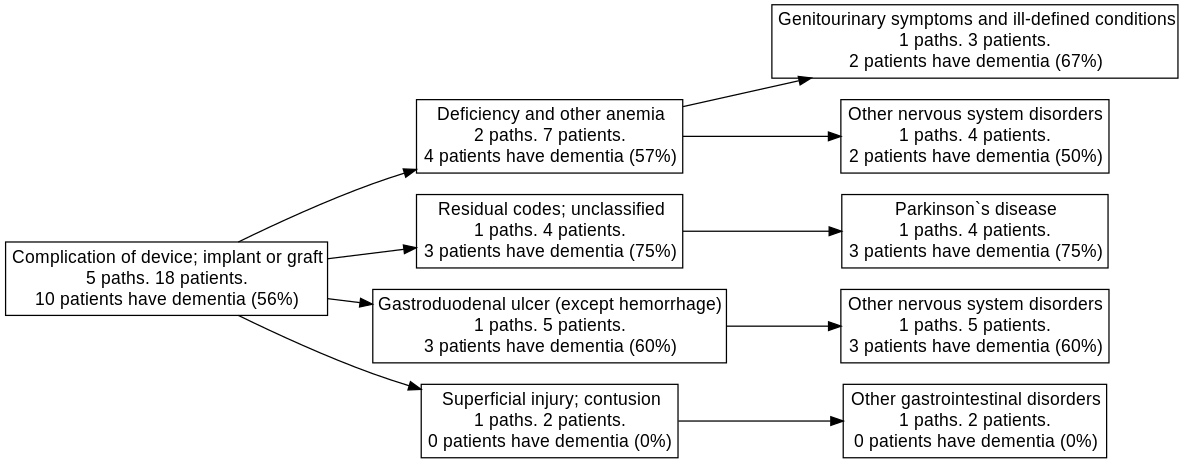


**Figure S1-41**


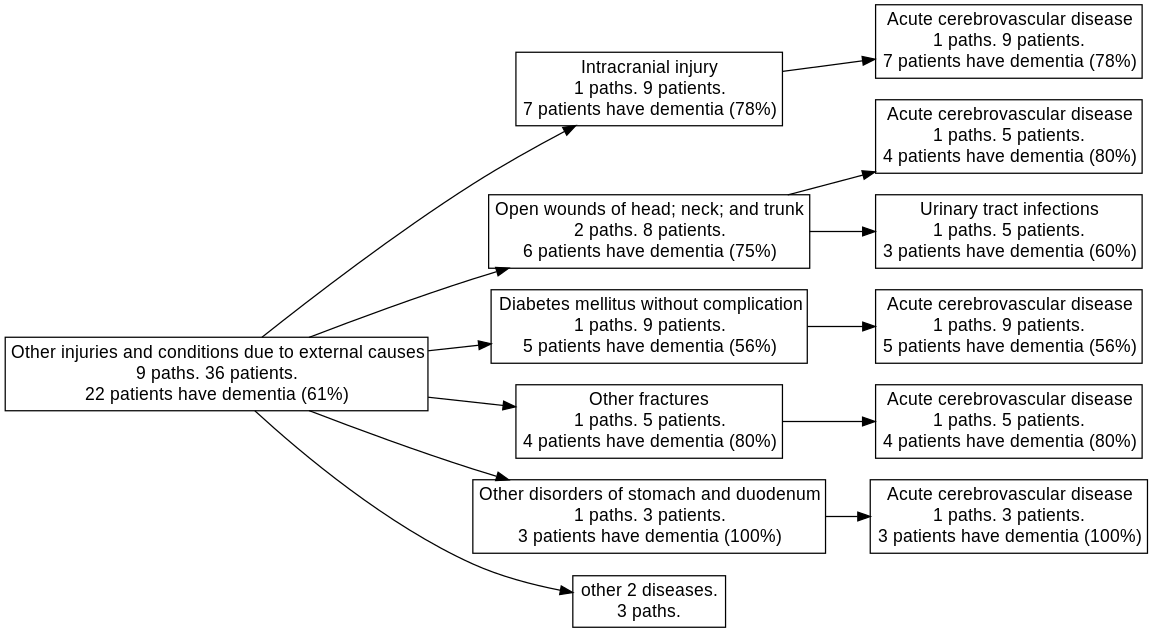


**Figure S1-42**


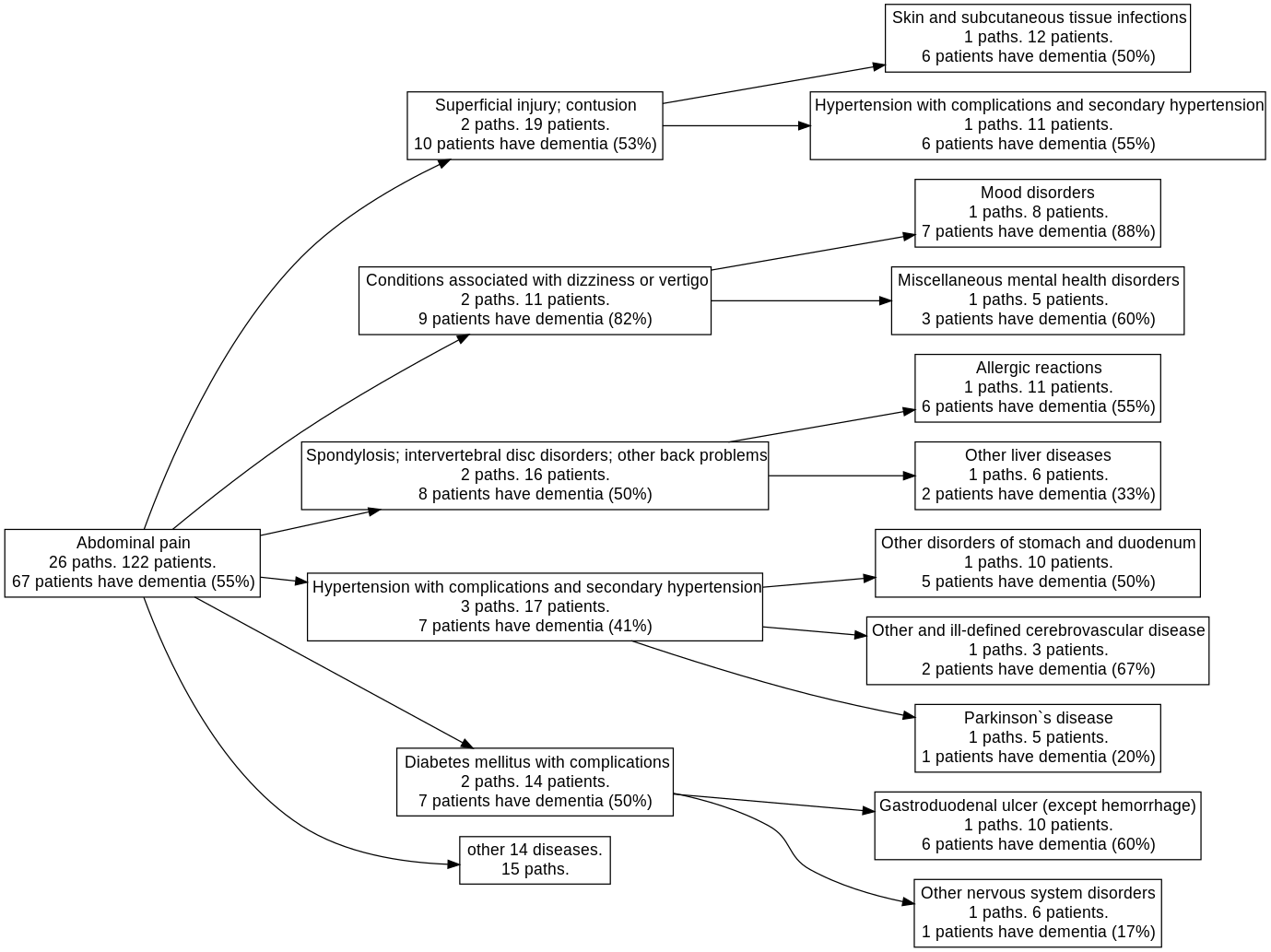


**Figure S1-43**


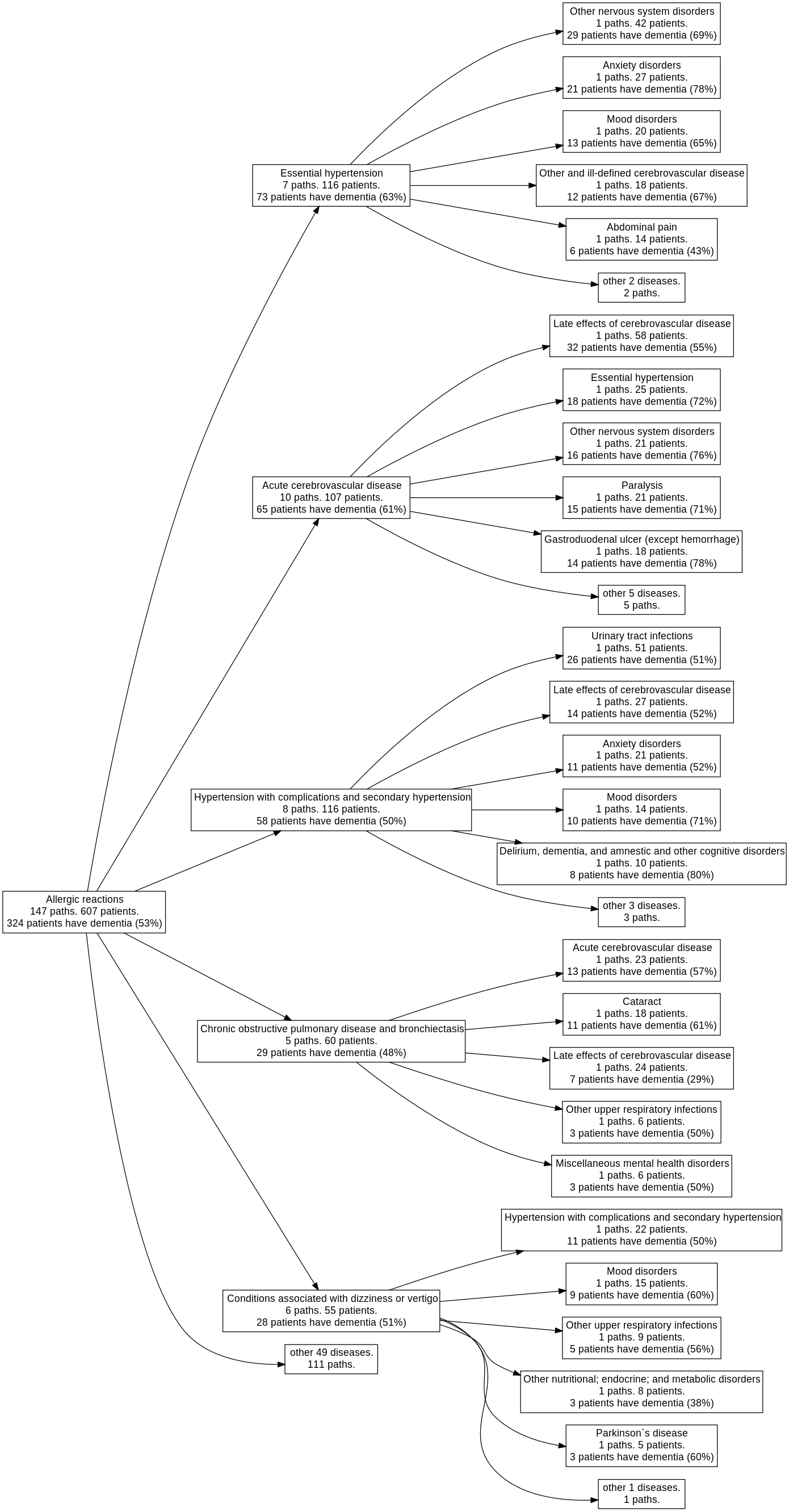


**Figure S1-44**


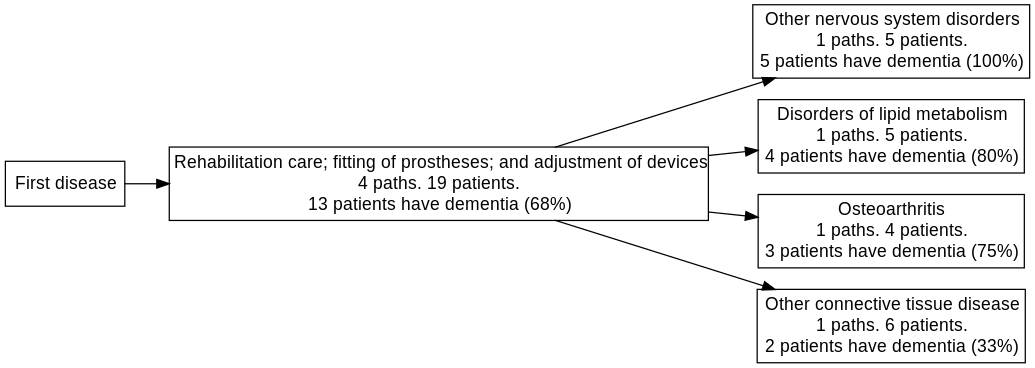


**Figure S1-45**


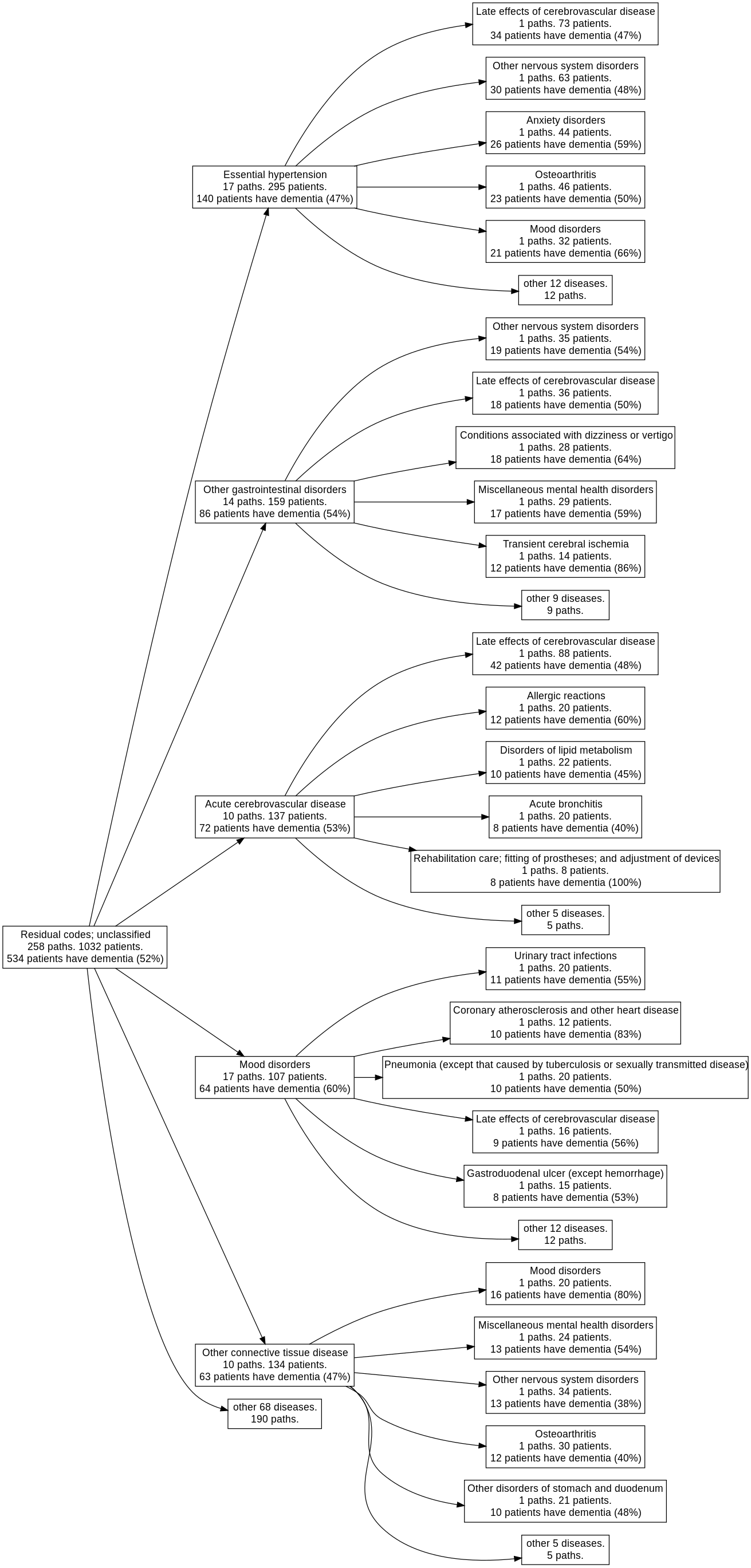


**Figure S1-46**


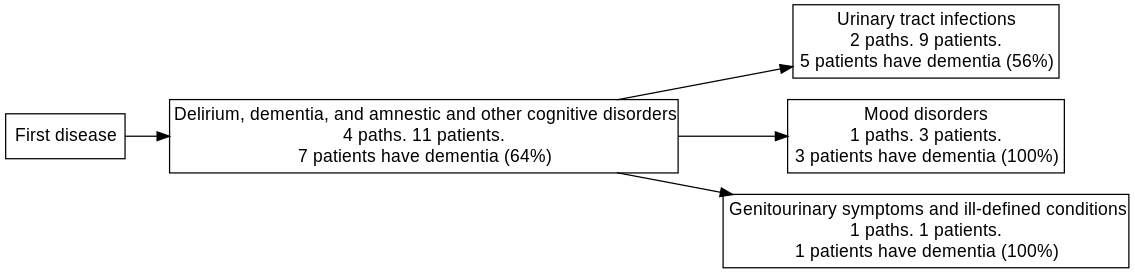


**Figure S1-47**


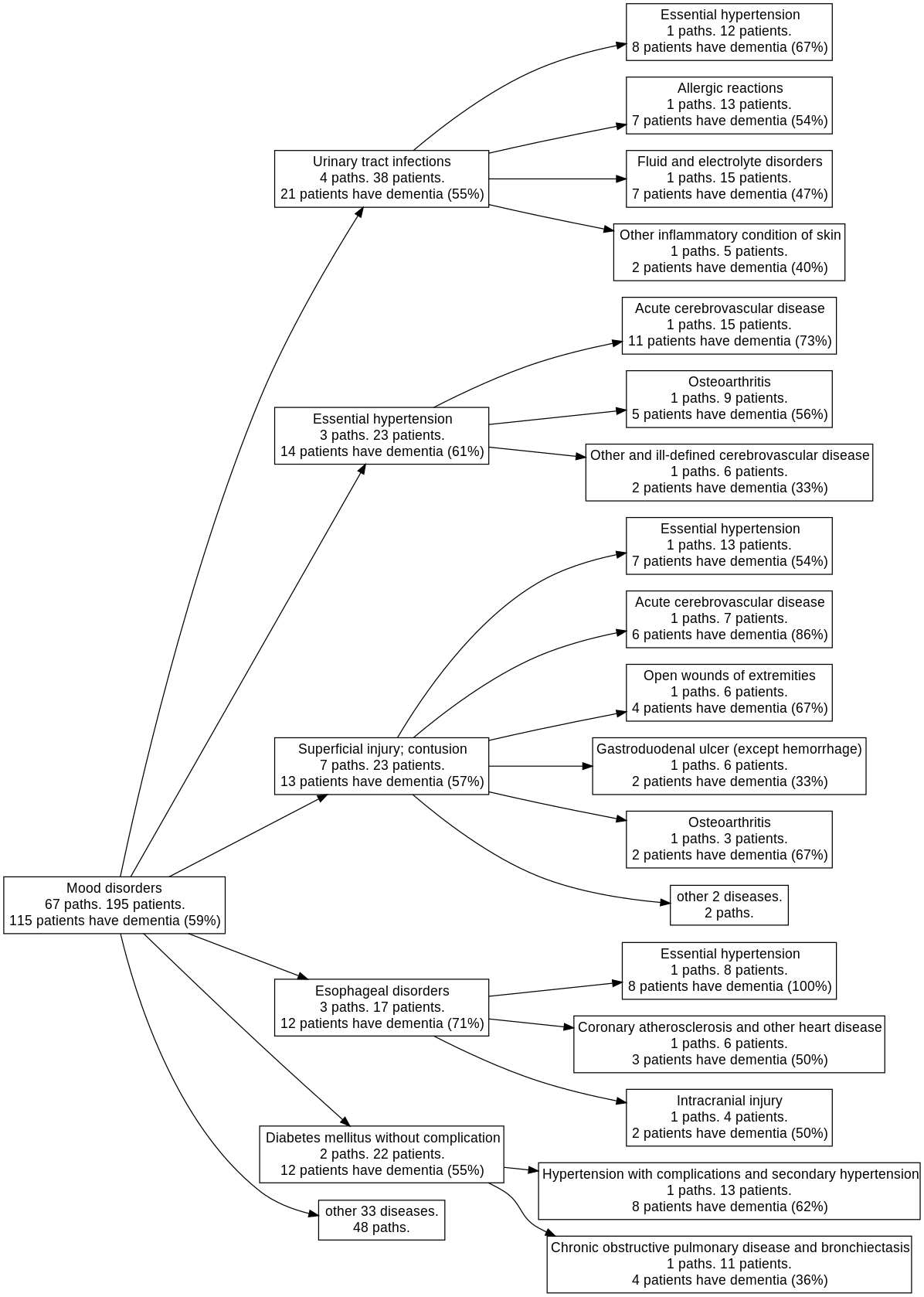


**Figure S1-48**


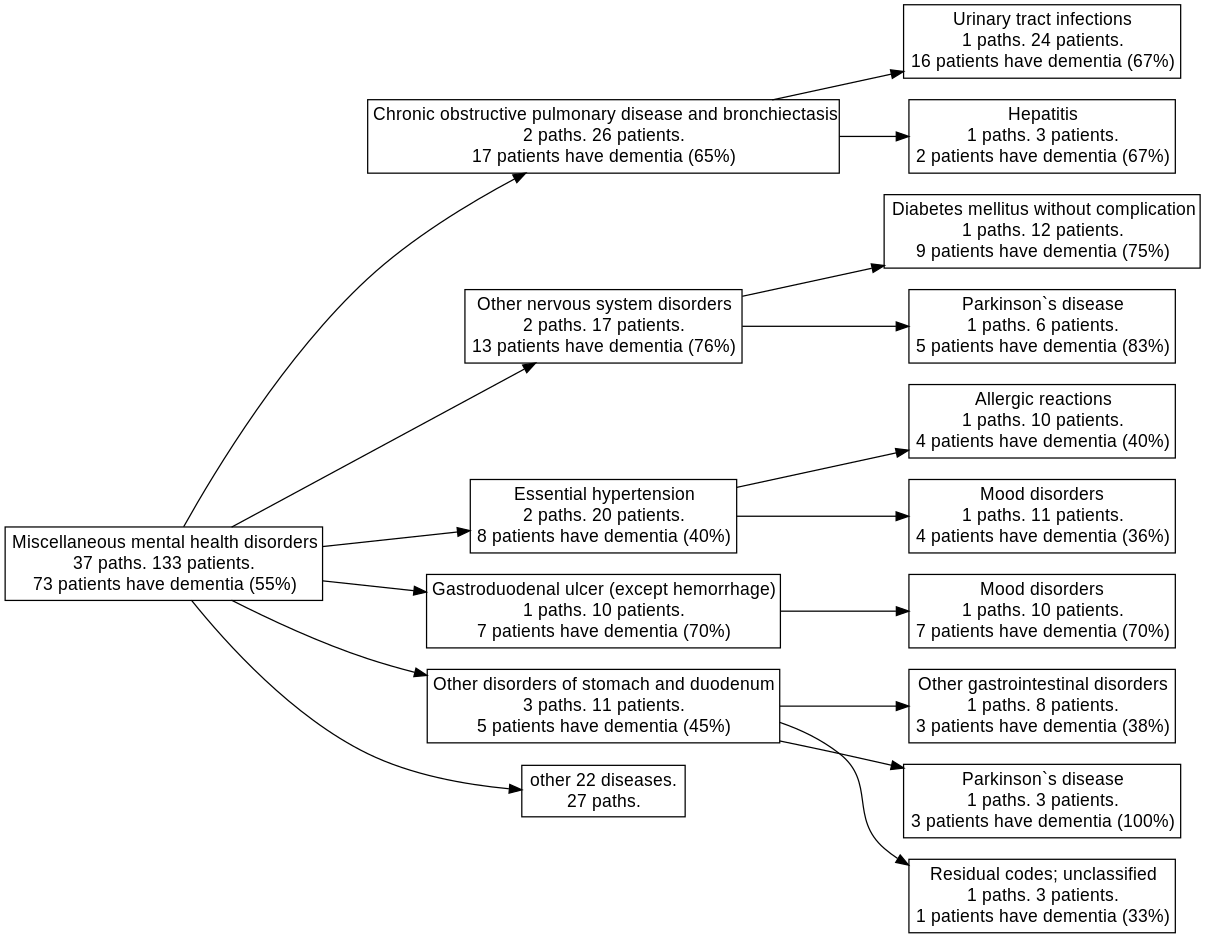

Supplement: Multimedia Appendix 1 [file jmir_v25i1e41858_app1.docx]
